# Dietary Partitioning in Two Co-occurring Caecilian Species (*Geotrypetes seraphini* and *Herpele squalostoma*) in Central Africa

**DOI:** 10.1093/iob/obz035

**Published:** 2019-12-31

**Authors:** M T Kouete, D C Blackburn

**Affiliations:** 1 California Academy of Sciences, San Francisco, CA 94118, USA; 2 Department of Natural History, Florida Museum of Natural History, University of Florida, Gainesville, FL 32611, USA

## Abstract

Trophic interactions among fossorial vertebrates remain poorly explored in tropical ecosystems. While caecilian species can co-occur, whether and how sympatric species partition dietary or other resources are largely unknown. Based on specimens collected during field surveys in southern Cameroon, we conducted a dietary analysis of two co-occurring caecilian species, *Geotrypetes seraphini* and *Herpele squalostoma*. We find a negligible overlap in the adult diets of these two species. Earthworms dominated the diet of adult *G. seraphini*, whereas we found that mole crickets were the most frequent prey items in adult *H. squalostoma*. The dietary breadth of adult *G. seraphini* is smaller than that of *H. squalostoma*, which consumes a variety of hard-bodied prey including mole crickets, cockroaches, beetles, and crabs. Juvenile diets were similar between these species and mostly contained earthworms and ants. We did not detect significant ontogenetic dietary shifts in either species, though adults generally consumed a broader diversity of prey. As adults, *G. seraphini* and *H. squalostoma* may partition prey categories by consuming soft-bodied and hard-bodied prey, respectively. Because most caecilians are likely opportunistic predators, we expect that sympatric species partition dietary resources either by preference for different soil layers or ability to consume different prey categories.

## Introduction

Among burrowing vertebrates, the ecology of caecilians—limbless and tropical amphibians—remains poorly studied (Wells 2007). Adults of most of the 213 species of caecilians are terrestrial, occupying leaf litter and soils in the tropics of Africa, Central, and South America, and southern Asia, and even oceanic islands such as São Tomé, the Seychelles, and the Philippines (Gower and Wilkinson 2008; AmphibiaWeb 2019). In addition, the free-living and feeding larvae of many caecilians are aquatic as are both larvae and adults of the South American Typhlonectidae. Many caecilians exhibit derived life histories and reproductive modes, including viviparity, direct development, extended parental care, and, perhaps most famously, dermatophagy, in which neonates are attended by the mother and feed on her skin (Wake and Dickie 1998; Wells 2007; Kupfer et al. 2008). Relative to salamanders and frogs, little is known of the diets of juvenile and adult caecilians. Most published accounts have limited sampling of intraspecific variation, including studies with just one or several individuals with no information on variation among individuals, sexes, or ontogenetic stages (e.g., Barbour and Loveridge 1928; Moll and Smith 1967; Presswell et al. 2002). Studies of caecilian diets that incorporate intraspecific variation are relatively recent, beginning largely with a series of quantitative studies on *Boulengerula taitana* (Gaborieau and Measey 2004), *Gegeneophis ramaswamii* (Measey et al. 2004), and *Schistometopum thomense* (Delêtre and Measey 2004). These works provide insights into the trophic niche breadth in caecilians and also demonstrate that these poorly known vertebrates are generalist predators of soil and terrestrial invertebrates (Gaborieau and Measey 2004).

While there are fewer than a dozen caecilian species for which intraspecific variation in diet has been studied, the dietary breadths of these species are fairly similar. Most studies reveal that caecilian diets are dominated by earthworms and aquatic or terrestrial soil insects such as termites and ants (e.g., Barbour and Loveridge 1928; Wake 1980; Verdade et al. 2000; Measey et al*.* 2004; Kupfer et al. 2005). Some species are known to consume hard-bodied invertebrate prey, including snails (Ngo et al. 2014) and crabs (Gudynas et al. 1988), and occasionally vertebrates such as scolecophidian snakes (Presswell et al. 2002), lizards (Moll and Smith 1967), and frogs (Gudynas et al. 1988; Kupfer et al. 2005). Hebrard et al. (1992) suggested that caecilians may be at least partially detritivorous, though later authors have rejected this (Delêtre and Measey 2004; Gaborieau and Measey 2004).

Most caecilians are thought to be dietary generalists with life history, ecology (i.e., aquatic vs. terrestrial), and seasonal changes in local prey abundance all driving variation within and among species (Kupfer et al. 2005; Ngo et al. 2014). Some caecilian species may specialize on particular prey types, including *Caecilia gracilis* (Maciel et al. 2012) and *Schistometopum thomense* (Delêtre and Measey 2004) which are both thought to specialize on earthworms. Because most studies sampled few individuals and neither prey abundance nor variation across sites or seasons is typically investigated, it is difficult to disentangle whether a species is a specialist or is instead an opportunist feeding on locally abundant prey types. In addition, almost nothing is known of the diets of caecilian species in sympatry, including whether co-occurring species might eat different prey. In the only study to address this issue, Jones et al. (2006) found differences in the diets of sympatric *Scolecomorphus vittatus* and *Boulengerula boulengeri* in Tanzania. While these species are found at the same sites, this study suggested that the two species feed on different types of earthworms related to foraging in different soil layers.

We extend dietary knowledge of caecilians by providing the second dietary study of co-occurring caecilians and the first studies of intraspecific variation between sexes and ontogenetic stages for two Cameroonian caecilian species, *Geotrypetes seraphini* (family Dermophiidae) and *Herpele squalostoma* (family Herpelidae). The caecilian fauna of Cameroon is the most phylogenetically diverse in Africa, comprising seven species in four families, including five endemic species (Wilkinson et al. 2011). In general, the diets of African caecilian species appear similar to that of species from other regions of the world, largely feeding on soil invertebrates (Table 1), but the diets of the diverse Cameroonian fauna have not yet been investigated. We focused our study on the two most common species, *G. seraphini* and *H. squalostoma*, in Cameroon. These species are widely distributed across West and Central Africa and can be especially abundant in cultivated areas such as gardens and plantations. In addition, these species are known to be sympatric in at least some parts of their respective ranges, including in Cameroon (Gower et al. 2015). While sometimes sympatric, the biology of each species is distinct, including in reproductive mode. *Herpele squalostoma* is oviparous with females attending eggs and juveniles, including provisioning young via skin feeding (Kouete et al. 2012, 2013). In contrast, *G. seraphini* is viviparous but also with altricial young provisioned by the attending mother by skin feeding (Parker 1956; O’Reilly et al. 1998). We conducted field surveys at three sites in Cameroon and examined gut contents of juveniles and adults of both species to (1) characterize the diversity of prey consumed (Delêtre and Measey 2004; Gaborieau and Measey 2004; Measey et al. 2004), (2) analyze patterns of intraspecific variation, both between sexes and ontogenetic stages (Kupfer et al. 2005; Jones et al. 2006; Kouete et al. 2012), and (3) test whether the dietary breadth and diversity of prey categories differ for these.

**Table 1:** Summary of prey categories in the diets of the nine African caecilian species studied to date, including members of the Dermophiidae (*Geotrypetes*, *Schistometopum*), Herpelidae (*Boulengerula*, *Herpele*), Indotyphlidae (*Sylvacaecilia*), and Scolecomorphidae (*Scolecomorphus*)

Study	Barbour and Loveridge (1928)	Jones et al. (2006)	Barbour and Loveridge (1928)	Jones et al. (2006)	Barbour and Loveridge (1928)	Gaborieau and Measey (2004)	Hebrard et al. (1992)	Barbour and Loveridge (1928)	This study	Delêtre and Measey 2004	Largen et al. (1972)	This study
Species investigated (sample size)	*Scolecomorphus uluguruensis* (*n=*?)	*Scolecomorphus vittatus* (*n *=20)	*Scolecomorphus vittatus* (*n *=7)	*Boulengerula boulengeri* (*n *=78)	*Boulengerula boulengeri* (*n=*2)	*Boulengerula taitana* (*n *=47)	*Boulengerula taitana* (*n *=14)	*Boulengerula uluguruensis* (*n=*?)	*Herpele squalostoma* (*n=*107)	*Schistometopum thomense*	*Sylvacaoecilia grandisonae* (*n *=6)	*Geotrypetes seraphini* (*n=*24)
Country	Tanzania	Tanzania	Tanzania	Tanzania	Tanzania	Kenya	Kenya	Tanzania	Cameroon	São Tomé	Ethiopia	Cameroon
Month	September–October 1926	June 2000–March 2001	October 1926	June 2000–March 2001	November–December 1926	May and December 2002; April 2003	July 1986; February 1989	September–October 1926	June–August 2014	October–November 2002	January 1971	June–August 2014
Prey categories												
Acari		X		X								
Arachnida										X		
Araneae				X								
Arthropod parts		X		X								
Blattoidea									X			
Coleoptera		X		X					X		X	
Chilopoda				X		X				X		
Coleoptera lavae				X								
Decapoda									X			
Dermaptera									X			
Diplura				X								
Diptera				X								
Diptera larvae				X								
Eggs of invertebrates		X		X					X			
Formicidae		X		X		X		X	X	X		X
Gastropoda						X						
Isopoda				X								
Isoptera	X			X	X	X	X	X	X			
Larvae of invertebrates							X		X	X	X	X
Lepidoptera larvae			X	X								
Neuroptera larvae						X						
Oligochaeta		X		X		X			X	X		X
Orthoptera									X			X
Ostracoda											X	
Schizomida		X		X								
Thysanoptera						X						
Tilupidae						X						

The most frequently encountered prey category is ants (Formicidae) whereas 15 prey categories were recorded in only one species. When known, sample size is indicated for each study. “X” indicates that the prey category was reported for that species in that study. Our prey categories of invertebrate larvae and invertebrate nymph were grouped together for comparability to other studies.

## Materials and methods

### Field surveys

**Fig. 1:**
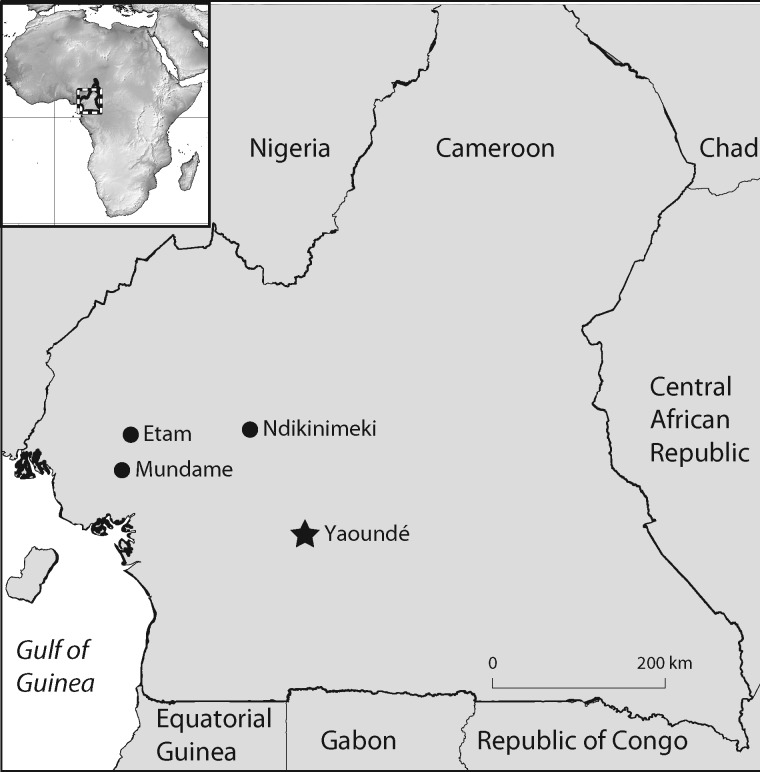
Distribution of the localities (Etam, Mundame, and Ndikinimeki) in Cameroon where caecilian specimens were sampled for this study.

We conducted our study at three field sites (Fig. 1) in southern Republic of Cameroon (datum WGS84): Etam (04°42′80.3″N, 09°32′52.7″E), Ndikinimeki (04°45′77.2″N, 10°48′25.9″E), and in Mundame near Meta quarter (04°33′08.5″N, 09°31′35.2″E). The habitat at these sites is modified by agriculture including both cash crops (cacao, coffee, rubber) and food crops (cassava, cocoyam, and other vegetables). We focused our searches near small streams <2 m wide as well as water seepages that, according to locals, flow only during the rainy season. We conducted our surveys in 2014 from mid-June to mid-August when the weather in Cameroon transitions from the minor rainy season into the minor dry season.

We collected caecilians by digging in soil with hoes and shovels (following Gower et al. 2015). Because stomach flushing is not known to be an effective tool for studying caecilian diets, we collected diet contents after preserving voucher specimens. We euthanized specimens within 24 h of their capture in an aqueous solution of MS-222 and then weighed (to the nearest grams) and measured (total length, to nearest millimeters) each specimen. A small sample of liver tissue was preserved in RNALater for future genetic studies. We then fixed all specimens in a solution of 10% neutral buffered formalin for ∼48 h, rinsed in water, and transferred them to 70% ethanol for storage. Once preserved, we took additional measurements for each specimen, including head length, head width (taken at the corner of the mouth), lower jaw length, and both width and circumference at mid-body. We used digital calipers to record all body measurements, except for the mid-body circumference which we measured using thread and a ruler (following Wilkinson et al. 2013). Following previous work by Malonza and Measey (2005) on another herpelid species (*Boulengerula taitanus*), we categorized specimens of both *G. seraphini* and *H. squalostoma* into three life stages based on total length: juveniles (<140 mm; containing neonates and larger individuals), subadults (<240 mm), and adults (>240 mm). We determined sex by direct examination of gonads via dissection; in some cases, gonads were not clearly discernable and thus we refrained from categorizing those specimens as male or female. All specimens are cataloged at the California Academy of Sciences (San Francisco, CA).

The occurrence and abundance of *G. seraphini* and *H. squalostoma* varied across our three field sites (Etam, Mundame, and Ndikinimeki). Two localities (Etam and Ndikinimeki) yielded sympatric populations of *G. seraphini* and *H. squalostoma.* During field sampling, individuals of both species co-occurred at three digging events including one in Etam and two in Ndikinimeki. At Ndikinimeni, we observed individuals as close as 10 cm suggesting that these two species can be closely associated. A total of 67 specimens of both species (including 4 *G. seraphini* and 63 *H. squalostoma*) were collected at Etam, whereas 24 others (comprising 21 *G*. *seraphini* and 3 *H*. *squalostoma*) were recorded at Ndikinimeki. Overall, we sampled 107 specimens of *H. squalostoma* (45 females, 38 males, 19 juveniles, and 5 indeterminate) and 24 specimens of *G. seraphini* (11 females, 7 males, 5 juveniles, and 1 indeterminate). The sex and/or ontogenetic stage could not be determined for several samples that are larger than juveniles (>140 mm) but that could not be identified definitively as either males or females. Statistics for length and mass of individuals of both species are summarized in Tables 2 and 3.

**Table 2:** Summary statistics for length and mass of juveniles of *G. seraphini* and *H. squalostoma*

	*G. seraphini*		*H. squalostoma*	
	Mass	Length	Mass	Length
	*n* = 5		*n* = 19	
Min	1.34	113	0.59	94
Max	1.94	135	1.36	136
Range	0.6	22	0.77	42
Med	1.7	125	0.99	111
Mean	1.65	125.2	0.94	113.1
SE	0.13	3.7	0.048	113
CI	0.36	10.25	0.1	2.54
Var	0.084	68.2	0.043	122.3
Std dev	0.3	8.26	0.21	11.1
CV	0.18	0.07	0.22	0.098

Min, minimum; Max, maximum; Med, median; SE, standard error on mean; CI, 95% confidence interval on mean; Var, variance; St dev, standard deviation; CV, coefficient of variation; *n*=sample size.

**Table 3:** Summary statistics for length and mass of adults and subadults of *G. seraphini* and *H. squalostoma*

	*G. seraphini*				*H. squalostoma*			
	Mass		Length		Mass		Length	
	Female (*n* = 11)	Male (*n* = 7)	Female (*n* = 11)	Male (*n* = 7)	Female (*n* = 45)	Male (*n* = 38)	Female (*n* = 45)	Male (*n* = 38)
Min	2.22	2.1	150	142	3.36	2.72	210	195
Max	25.7	12.4	326	265	41.09	30.27	463	424
Range	23.48	10.3	176	123	37.73	27.55	253	229
Med	17.48	6.9	285	211	7.48	8.29	286	279
Mean	17.07	7.3	248.45	207.86	9.1	9.46	287.3	285.68
SE	1.89	1.4	19.84	15.30	0.95	0.82	7.82	7.29
CI	4.21	3.4	44.21	37.45	1.92	1.65	15.8	14.78
Var	39.28	13.2	4330.67	1639.48	41	25.27	2754.5	2021.9
St dev	6.27	3.6	65.81	40.49	6.4	5.03	52.5	44.97
CV	0.37	0.5	0.26	0.19	0.7	0.53	0.18	0.16

min, minimum; max, maximum; med, median; SE, standard error on mean; CI, 95% confidence interval on mean; var, variance; st dev, standard deviation; CV, coefficient of variation; *n*=sample size.

### Categorization of diet

We incised preserved specimens ventrally from below the heart to just anterior to the cloaca. We removed and weighed on a Pesola scale the alimentary canal (i.e., gut) and weighed its contents separately (to the nearest 0.0001 g). We sorted gut contents using a dissecting microscope, and then counted and identified individual prey items (generally to the order or family, but when possible to the genus level). When counting, we attempted to avoid overestimating the number of prey that might be represented by multiple fragments, generated in part during feeding when caecilians bite and then spin to tear prey items (Measey and Herrel 2006). Especially for earthworms, we searched among fragments in the contents of an individual to attempt matches and then treated these co-occurring pieces as a single item. We were unable to identify taxa of earthworms more specifically due to the fragmentary nature of these prey items. Subsequent data, including length and width for whole food items, were recorded using an ocular micrometer with a Leica S6D microscope (10× magnification), aided by a handheld ruler for large prey; we did not record these data for partial or dissolved food items. In cases where partially digested food items were recognizable (i.e., head capsules of ants and orthopterans), we estimated dimensions following Hirai and Matsui (2001). Both direct and estimated measurements of length and width were used to evaluate the volume of food items by applying the equation of ellipsoid bodies (Colli and Zamboni 1999), V=43π×(12×length)×(12×width)2. When searching through gut contents of juveniles of both *G. seraphini* and *H. squalostoma*, we carefully inspected various food items to determine whether skin fragments ingested during skin-feeding might be attached or embedded. We unambiguously identified skin fragments by staining these with iodine. The lipids in the skin fragments become a vivid yellow color when exposed to iodine (Wilkinson et al. 2008). Because of the irregularity of their size and shape, we only counted the frequency of skin fragments among specimens rather than counting and measuring individual pieces within an individual. We grouped other measurable items (with values for length and width) into food categories and used these to quantify the diet of both *G. seraphini and H. squalostoma*.

### Statistical analyses

We evaluated both the frequency and abundance (taking into account the number and the volume) of food found in the gut of specimens of each species and at different life stages. To determine the relative importance of each food category, we calculated the index of relative importance (IRI) following Pinkas et al. (1971) for juveniles and both males and females (for subadults and adults combined) of each species. When calculating IRI, we considered only individuals in which the gut contained at least one prey item; individuals with empty guts were excluded from analyses. For any food category i, we calculated IRI as follows:

${\mathrm{IRI}}_{i}=({\mathrm{NP}}_{i}+{\mathrm{VP}}_{i})\times {\mathrm{FP}}_{i}$, where ${\mathrm{FP}}_{i}$ is the percentage of occurrence of food items category i (100 × number of individuals containing food items category i/total number of individuals), ${\mathrm{NP}}_{i}$ is the percentage abundance (100 × total number of food items i contained in guts of all individuals/total number of items for all food categories contained in all individuals), and ${\mathrm{VP}}_{i}$ is the volumetric percentage (100 × total volume of food items category i in guts of all individuals/total volume of all food categories in all individuals).

To make comparisons among species, sex, and ontogenetic stages, we calculated dietary breadth (B) following Levins (1968): $=\frac{1}{\sum_{N}P_{j}^{2}}$, where $P_{j}$ is the numerical proportion of prey category j in the diet and N is the total number of prey categories. Values of B range from 1 to N depending on whether only one prey category or all food categories occurred in a group of individuals.

We also evaluated the extent of overlap in diet among species, sexes, and ontogenetic stages. We calculated an index of overlap, PS, proposed by Schoener (1968). For specimens belonging to group i, PS was determined as: ${\mathrm{PS}}_{i}=1\;-\;0.5\sum_{j}\left|P_{ij}-q_{j\;}\right|$, where $P_{ij}$ is the numerical proportion of food category j in group *i*’s diet, and $q_{j}$ is the proportion of diet category j recorded in all animal groups considered. Values of PS range from 0 (no overlap) to 1 (complete overlap).

Because variation within a species can complicate comparisons between species (Sevenster and Bouton 1997), we took several approaches to investigate variation in diet among individuals. First, we used Mantel tests to examine the extent of diet both between the two species and between subadults and adults (considered together) and juveniles within each species. To compute the Mantel test (a non-parametric test that evaluates correlation among two matrices), we followed Luo and Fox (1996) because their method addresses several challenges inherent to analysis of dietary data, such as unequal sampling sizes within and among species as well as abundances and frequencies that are often aggregated (Anderson 2001; see Guillot and Rousset [2013] for possible biases of simple and partial Mantel tests). This method requires construction of a distance matrix that represents the overlap of diet between two groups and a second matrix that represents the null hypothesis of perfect segregation of diet between the two groups. We calculated the distance matrix from the proportion of food items in the gut of each individual in each group compared. For this, we averaged food categories across all individuals by dividing the volumetric proportions by the total volume of each prey category (Bolnick et al. 2002). We used these proportions to calculate the Manhattan distance, an index relative to the proportional similarity measure (see Luo and Fox 1996). We slightly modified this matrix of similarities to correct for unequal sample size that provides more power to our test (following Luo and Fox 1996). All Mantel tests were computed using the *ape* package (Paradis et al. 2004) in R version 3.3.2 (R Core Team 2016) with each analysis set to run for 1000 permutations. To investigate differences in diet that may be due to differences in habitat type (coffee vs. food-crop farms), we used the Fisher’s exact test for chi-square. Last, we used ANCOVA to investigate differences of gut content mass (dependent variable) that may be due to sex (interactions males/females × gut content mass). Gut content mass for each specimen was calculated as a sum of the masses of individual prey items consumed. We used the cor.test function to explore relationships between the number of prey or their size (length and width) and body attributes in adult *G. seraphini* and *H. squalostoma*.

To perform ANCOVAs, we determined the best predictor for gut content mass from a subset of the body attributes recorded for each species (total length, head length, head width, lower jaw length, mid-body width, mid-body circumference). We used this approach so as to analytically choose the independent variable for ANCOVA rather than choosing one based on other assumptions (e.g., Measey et al. 2004; Jones et al. 2006). Using the set of predictor variables, we constructed a generalized linear model and performed a multi-model selection using the “dredge” function in R’s MuMIn package (Bartoń 2012). This function constructs and fits all possible candidate sub-models nested within the global model (comprising all recorded predictors), and then ranks them according to either model averaging or any other specified information criteria). We used the Bayesian Information Criterion (BIC) to rank our candidate models (Johnson and Omland 2004). We considered the best-fitting model to be the model with the lowest BIC score, but also considered models within 2 BIC units of the best-fit model. For cases in which the best models with the dredging approach comprised more than one covariate, we additionally performed ANOVA to pick the ultimate best predictor for gut content mass.

We assessed data normality by applying the Shapiro test. We natural-log transformed gut content mass—the dependent variable in our analysis—because it did not meet the assumption of normality. We further compared the diets of female and male *G. seraphini* and *H. squalostoma* by assessing the relationships of the independent variable for each species as a function of log-transformed values of gut content mass. We used a significance cut-off of 0.05 for statistical tests, all of which we performed in R (R Core Team 2016).

## Results

### Gut contents

Our analysis of gut contents produced 10 prey categories (Table 4) that differ in their frequency, abundance, and/or volume across species, life stage, and sex (Table 5). The dominant prey category differs between species for adults and subadults (Table 4), mostly due to the amount of earthworms and crickets consumed by each species. Earthworms constituted the most frequent (54%) and most abundant (54%) prey in the diet of *G. seraphini* whereas mole crickets were the most frequent prey (42%) and ants the most abundant (34%) in the diet of *H. squalostoma*. Most specimens of *H. squalostoma* contained a single cricket. In contrast to *H. squalostoma*, the cricket consumption of *G. seraphini* was low (8%). Instead, the most important prey category for *G. seraphini* was earthworms, representing 80% of all prey volume consumed by this species. Earthworms dominate the diet of adult *G. seraphini*, but *H. squalostoma* consumed significantly more of both earthworms and ants (*x*^2^ = 0.29, *P *<* *0.001, df = 756, two-tailed test). Total number of prey categories consumed also differs between the species, with *G. seraphini* consuming fewer prey types than *H. squalostoma*. The more taxonomically diverse diet of *H. squalostoma* leads to a larger dietary breadth (4) in comparison to *G. seraphini* (2.5), as well as low overlap between adults of these two species (PS = 0.31 and PS = 0.47 using prey volume and prey frequencies, respectively). The Mantel test indicate that there is a significant low overlap (observed = 34; two-tailed *t*-test *P *=* *0.003) between adults of *G. seraphini* and *H. squalostoma*.

**Table 4:** Characterization of the diets of males and females of *Geotrypetes seraphini* and *Herpele squalostoma*

	*G. seraphini* (6 females, 4 males)												*H. squalostoma* (38 females, 35 males)											
	*F*%		*N*%		*V*%		%IRI		Mean				*F*%		*N*%		*V*%		%IRI		Avg			
Prey categories	F	M	F	M	F	M	F	M	Nm	Nf	Vm	Vf	F	M	F	M	F	M	F	M	Nm	Nf	Vm	Vf
Formicidae (ants)	50	25	33.3	25	0.5	5.1	17.7	5.6	0.25	0.5	0.16	0.32	36.84	34.3	26.2	39.3	0.44	11.8	8.5	19.5	3.43	1.26	23.06	0.71
Coleoptera (beetles)	0	0	0	0	0	0	0	0	0	0	0	0	15.8	0	4.37	0	1.86	0	0.85	0	0	0.21	3	0.02
Decapoda (crabs)	0	0	0	0	0	0	0	0	0	0	0	0	0	5.7	0	0.65	0	0.96	0	0.1	0.06	0	1.88	0
Dermaptera (earwigs)	0	0	0	0	0	0	0	0	0	0	0	0	0	2.86	0	2.95	0	0.23	0	0.1	0.26	0	0.46	0
Eggs of invertebrates	0	0	0	0	0	0	0	0	0	0	0	0	18.4	20	39.3	37.4	0.06	6.3	6.3	9.7	3.26	1.9	12.32	0.1
Blattoidea (cockroaches)	0	0	0	0	0	0	0	0	0	0	0	0	2.63	5.7	1.09	0.98	0.03	0.08	0.02	0.06	0.09	0.05	0.16	0.05
Orthoptera (crickets)	16.7	0	11.1	0	41.5	0	9.2	0	0	0.17	0	28.3	84.2	74.3	23	8.5	90.5	64.2	82.6	60.1	0.74	1.1	126	148.72
Invertebrate nymphs	16.7	0	11.1	0	0.95	0	2.1	0	0	0.17	0	0.65	10.5	8.57	2.2	3.9	2.7	0.23	0	0.4	0.34	0.1	0.45	4.3
Isoptera (termites)	0	0	0	0	0	0	0	0	0	0	0	0	0	2.86	0	1.31	0	0.03	0	0.04	0.11	0	0.06	0
Oligochaeta (earthworm)	66.7	75	44.4	75	57.2	94.9	71	94.4	0.75	0.7	3	39	18.4	42.9	3.83	4.9	5.12	16.2	1.4	10.06	0.43	0.18	31.78	8.17

Distribution of frequency *F*%, abundance *N*%, volume *V*%, and index of relative importance (IRI; expressed as a percent prey) for females (F) and males (M). The table comprises only specimens that guts contained at least one identifiable prey item. Mean prey number (N) and volume (V) for females (Nf, Vf) and males (Nm, Vm) is indicated.

**Table 5:** Average total prey number and volume per species and life stage in *G. seraphini* and *H. squalostoma*

	*G. seraphini* (*N* = 5)		*H. squalostoma* (*N* = 19)	
Life stage/sex	Average total prey number	Average total prey volume (mL)	Average total prey number	Average total prey volume (mL)
Juveniles	1.8±0.8	5.6±4.4	1.0±0.0	2.9±1.7
Females	1.0±0.0	94.8±49.3	2.6±0.6	88.3±10.3
Males	1.0±0.0	0.2±0.2	2.6±0.6	99.6±14.3

The dredging approach to select the best predictor of gut mass resulted in different predictors for each species. For *G. seraphini*, the best model had the lowest BIC score, >1 BIC unit lower than the next best-fitting model, and contained mid-body circumference as a covariate (Supplementary Tables S1 and S2); the two next best-fitting models contained one additional parameter each. For *H. squalostoma*, the best model contained just one covariate, total length and had the lowest BIC, which was >1 BIC unit lower than the next best models (Supplementary Table S3).

### Sex-based dietary differences

**Fig. 2:**
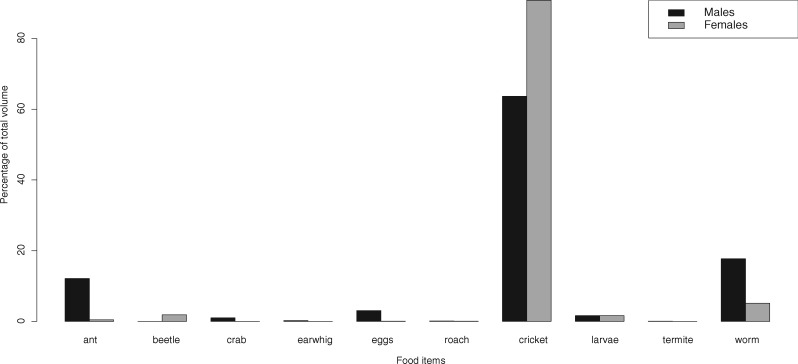
Prey categories consumed by female and male *Herpele squalostoma*.

**Fig. 3:**
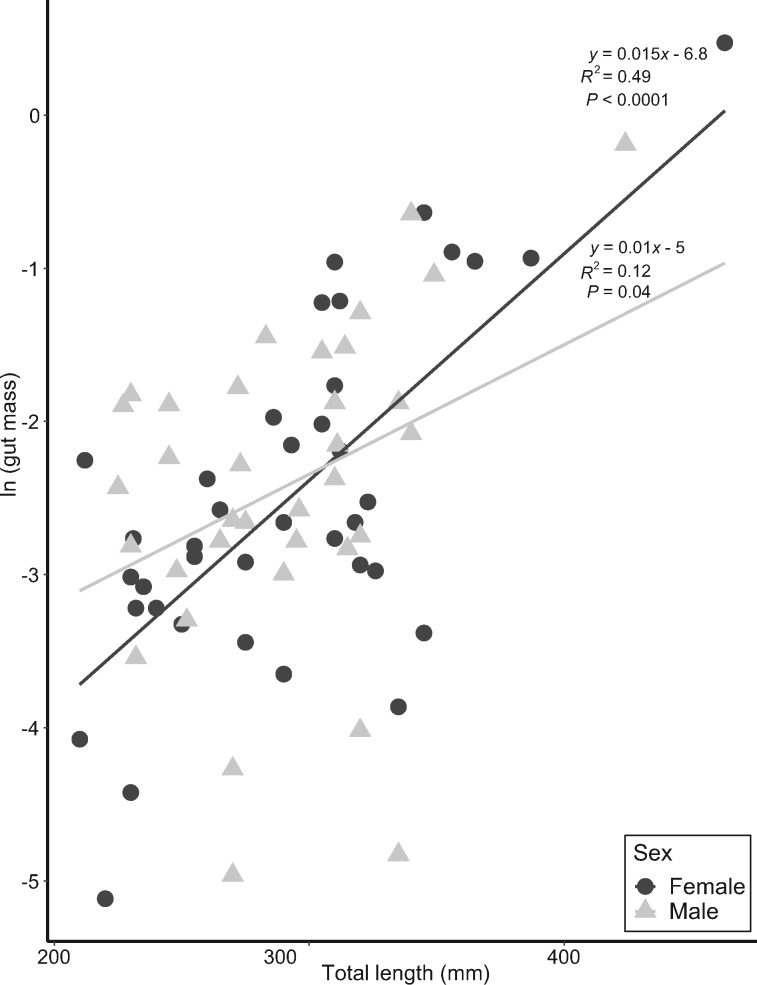
Regression of mass of gut content (in g) as a function of total length (TL) (in mm) between female and male *Herpele squalostoma*. Mass of gut content was natural log-transformed to meet the assumption of normality.

In *H. squalostoma*, neither size nor sex influenced prey type. While some prey items occurred in only one sex, there were no significant differences between males and females in overall volumetric proportions of prey items consumed by males and females (Fig. 2) or dietary breadths (females 3.6, and males 3.3; PS* *= 0.7). In general, there was a significant and positive relationship for both sexes between body size and prey mass (Fig. 3 and Table 6), and the interaction of sex * size was not significant (*F*_1,__69_ = 1.95, *P *=* *0.17, Table 7). Five (one male and four female) specimens of *H. squalostoma* contained only a small amount, if any, of prey (≤0.001 g). These four females were of an adult size (>240 mm) typically associated with high levels of prey consumption (see above). One was found attending a litter of five young and a second (with 0.051 g of prey items) was found brooding a clutch of eggs.

**Table 6:** Log-transformed relationships of the dependent (gut mass) and the independent variables for the diets of females and males *G. seraphini* and *H. squalostoma*

Species	Independent variable	*y*-Intercept	Slope	Standard error of slope	Correlation coefficient
*G. seraphini*	MBC	−5.14	0.11	0.08	0.17
		(−4.81)	(0.038)	(0.11)	(0.03)
*H. squalostoma*	TL	−6.83	0.015	0.0025	0.49
		(−4.90)	(0.009)	(0.0039)	(0.12)

Values for males are in parentheses. Significant relationships are in bold. MBC, mid-body circumference; TL, animal total length.

**Table 7:** Summary of ANCOVAs of gut content mass (the dependent variable) as a function of animal total length (independent variable) for *H. squalostoma*

Sources	Df	Sum of square	*F*	*P*-value
TL	1	26.36	31.9	**<0.0001**
Sex	1	0.19	0.22	0.64

Significant *P-*value is indicated in bold. The interaction TL * Sex was not significant.

**Fig. 4:**
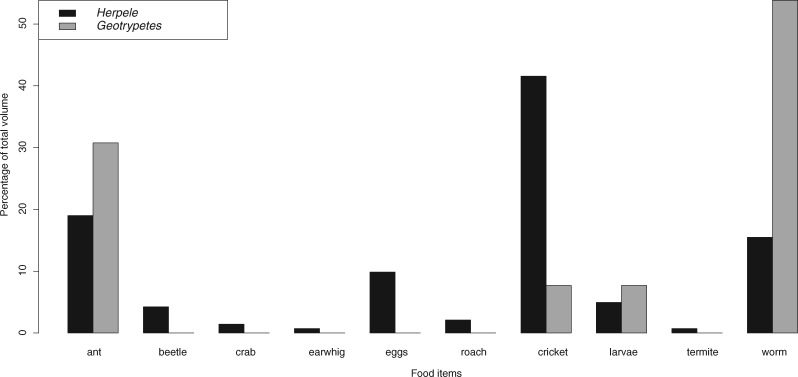
Prey categories consumed by adult *G. seraphini* and *H. squalostoma*.

**Fig. 5:**
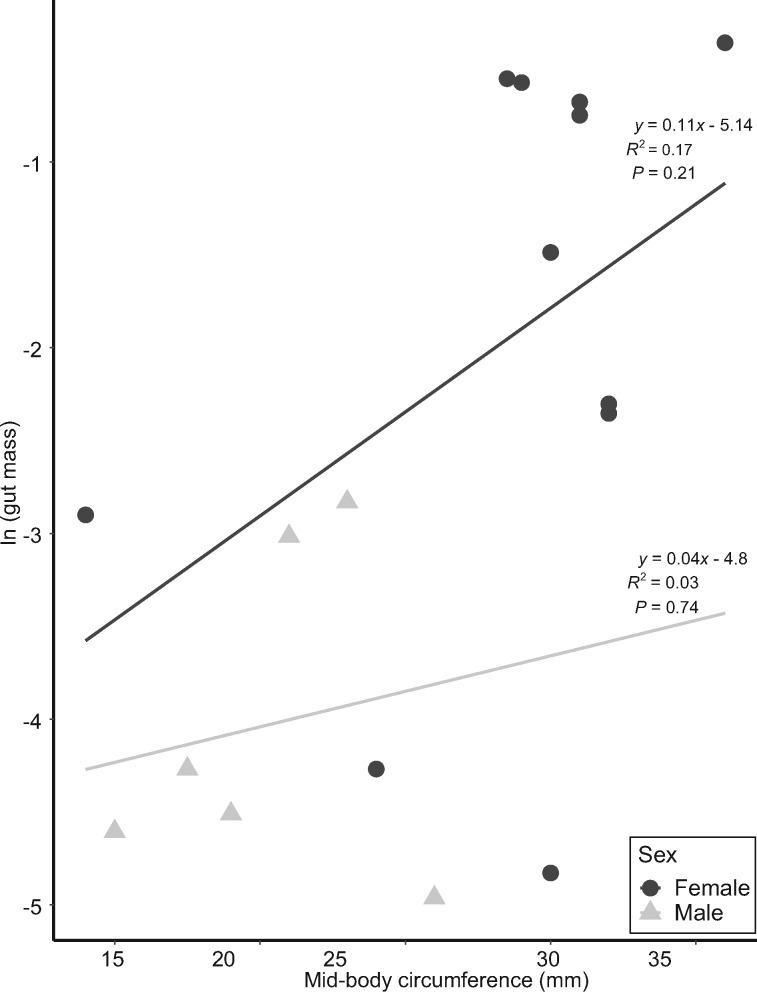
Regression of mass of gut content (in g) as a function of MBC (in mm) between female and male *Geotrypetes seraphini*. Mass of gut content was natural log-transformed to meet the assumption of normality.

Male and female *G. seraphini* had similar diets with earthworms dominating the diets of both sexes (Fig. 4). As with *H. squalostoma*, prey consumption was explained by body size. Neither sex nor the interaction of sex * size were significant, although this may change with greater sample sizes (Tables 6 and 8 and Fig. 5).

**Table 8:** Summary of ANCOVAs of gut content mass (the independent variable) as a function of MBC (the dependent variable) for *G. seraphini*

Sources	Df	Sum of square	*F*	*P*-value
MBC	1	17.6	9.4	**<0.009**
Sex	1	3.7	2	0.18

Significant *P-*value is indicated in bold. The interaction MBC * Sex was not significant.

### Diets of juvenile caecilians

Juvenile specimens of *G. seraphini* (*n* = 5) and *H. squalostoma* (*n* = 19) contained only two prey categories: ants and earthworms (Table 9). Ants were encountered more frequently across individuals and were the most abundant prey item, whereas earthworms represented the greatest volume (Table 9). This pattern is consistent for juveniles both within and between these two species. Juveniles of both species exhibited similar dietary breadths (1.5 for *G. seraphini*, 2 for *H. squalostoma*) and their diets largely overlapped (overlap of 0.8).

**Table 9:** Prey categories by juvenile *G. seraphini* and *H. squalostoma*

Prey categories	*G. seraphini* (*N* = 5)				*H. squalostoma* (*N* = 19)			
	*F* (%)	*N* (%)	*V* (%)	%IRI	*F* (%)	*N* (%)	*V* (%)	%IRI
Formicidae	60	78	9	**53.9**	61	61	14	48.4
Oligochaeta	40	22	91	46.1	39	39	86	**51.6**

*F*, *N*, and *V* are, respectively, the frequency of the abundance and the volume (expressed in percentage) of each prey category. IRI is calculated as indicated by Pinkas et al. (1971) and expressed in percent. The dominant prey category for each species is in bold.

**Fig. 6:**
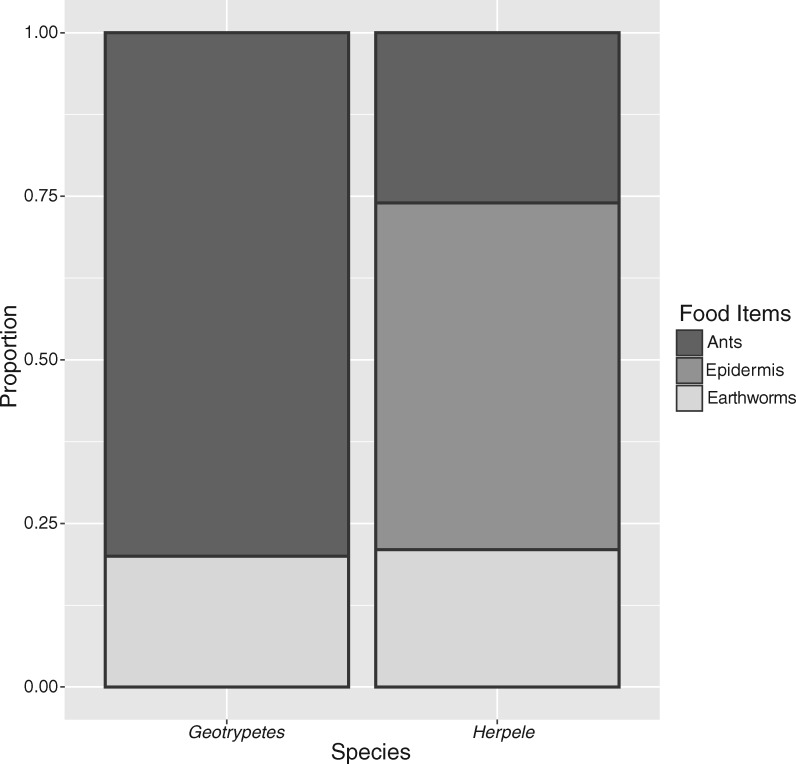
Food categories consumed by juveniles of *G. seraphini* and *H. squalostoma*, represented as numerical proportion of total food categories. Note the inclusion of skin fragments in the diet of juveniles of *H. squalostoma* alters the percentages of food items reported in Table 9, which includes only prey items.

Juvenile diets of the two species differed primarily in the presence of skin (Fig. 6). Most (53%) juvenile *H. squalostoma* contained skin fragments with some (21%) containing only skin fragments, most likely reflecting dermatophagy while attended by mothers. Specimens of *H. squalostoma* that contained skin fragments varied in size from 103 to 180 mm in length. The largest specimen (180 mm) that contained skin fragments is larger than the length at which we considered specimens to be adult, and contained only a large mass of skin fragments (mass = 0.018 g) in its gut. The smallest juvenile specimens of *H. squalostoma* (94 and 97 mm in length, respectively) did not contain skin fragments, but contained earthworms or both earthworms and ants. One juvenile *H. squalostoma* contained skin fragments as well as earthworms and ants.

### Ontogenetic dietary analysis

Earthworms dominated the diets of both juvenile and adult *G. seraphini*. The diet of adults differed from juveniles by adding both crickets and invertebrate larvae to the juvenile diet of ants and earthworms, as well as adding larger preys. The adult diets included considerably larger earthworms; the maximum earthworm size recorded for adults was 35 and 3 mm, for length and width, respectively, in contrast to 12.2 and 2 mm in juveniles. Adult *G. seraphini* consumed fewer and larger prey items than juveniles, with body length being negatively correlated with prey number (Table 10). The maximum number of prey items in a single juvenile *G. seraphini* was five (all ants) but the maximum recorded in an adult was only three (ant, cricket, and invertebrate larva). Mean prey volume increased from juvenile to adult, whereas mean prey number decreased (Tables 4 and 5). We did not detect a significant relationship between any body attributes (length, head width, head length, and lower jaw length) and prey size (length and width) (Table 10). A Mantel test revealed no statistically significant ontogenetic diet shift for *G. seraphini* (observed = 12; two-tailed *t*-test *P *=* *0.11).

**Table 10:** Relationship of prey size (length and width) and body attributes of *Geotrypetes seraphini* and *Herpele squalostoma*

Body attributes/prey	*G. seraphini*			*H. squalostoma*		
attributes	Correlation coefficient (*r*)	Correlation equation	*P*-value	Correlation coefficient (*r*)	Correlation equation	*P*-value
Length/prey number	−0.08	1.7–0.001*x*	0.70	0.3	−10.7+0.06*x*	**0.004**
HW/prey width	0.30	0.02+0.36*x*	0.30	0.5	0.1+0.7*x*	**<0.001**
HL/prey length	0.40	−21+4.2*x*	0.09	0.4	−15.6+4.1*x*	**<0.001**
LJL/prey length	0.25	−24.1+5.2*x*	0.06	0.4	−13.1+4.5*x*	**<0.001**

PN, prey number; HW, head width; HL, head length; LJL, lower jaw length. Significant *P-*values are indicated in bold.

The dietary shift from juvenile to adult *H. squalostoma* is similar to that observed in *G. seraphini*. The number of prey categories increased from two in juveniles to seven in adult *H. squalostoma*, including several hard-bodied prey that dominated the diet of adults such as beetles, cockroaches, crabs, and crickets. The most abundant prey in both females and males were invertebrate eggs and ants (Table 4). For both sexes, mole crickets were both the most frequently encountered in females and males (84.2% and 74.3%, respectively) and comprised the largest prey volume (90.5% and 64.2%; Table 4). Crickets may be among the first prey categories added to the juvenile diet in *H. squalostoma*; the smallest specimen with a cricket was 185 mm long. Mean prey volume increased from juvenile to adult as did, in contrast to *G. seraphini*, mean prey number (Table 5). There is a positive significant correlation between prey size (length and width) and body attributes in juvenile and adult *H. squalostoma* (Table 10). A Mantel test revealed no statistically significant ontogenetic diet shift for *H. squalostoma* (observed = 87.7; two-tailed *t*-test *P *=* *0.56).

## Discussion

### Diet of sympatric caecilians

Our work reveals important differences in the diets of two co-occurring Central African caecilians. Adults of both *G. seraphini* and *H. squalostoma* consumed earthworms, ants, crickets, and various invertebrate larvae. However, the relative importance of these prey categories and the dietary breadth differed with the diet of *G. seraphini* being relative narrow (breadth = 1.5) and dominated by earthworms and that of *H. squalostoma* being substantially broader and dominated by mole crickets (breadth = 3.5). The greater dietary breadth of *H. squalostoma* includes six additional prey categories (invertebrate eggs, beetles, cockroaches, crabs, earwigs, and termites) and contributes to the low but still significant overlap in diet (PSI = 0.46) between these two species. A previous study by Jones et al. (2006) on two caecilian species that occur in sympatry in East Africa (at Nilo Forest Reserve in Tanzania) found similar patterns related to breadth and relative importance of difference. In this case, *Scolecomophus vittatus* (family Scolecomorphidae) had a relatively narrow diet in comparison to the co-occuring *B. boulengeri* (family Herpelidae). Earthworms dominate the diets of both species, though *S. vittatus* consumed mostly large, pigmented epigeic species and *B. boulengeri* consumed smaller, unpigmented endogeic species. While Jones et al. (2006) did not calculate statistics for dietary breadth and overlap, it is clear that in both our study and theirs that the sympatric caecilian species differ in both breadth and prey categories.

The different diets of adult *G. seraphini* and *H. squalostoma* are suggestive of partitioning dietary resources by microhabitat. First, earthworms consumed by *G. seraphini* are large and pigmented (typical of epigeic taxa) suggesting that this species forages at or near the surface whereas the mole crickets that dominate the diet of *H. squalostoma* suggest this species forages underground. Mole crickets are active burrowers that create tunnels in which they lay and guard eggs (Bennet-Clark 1987), and often considered agricultural pests (Brandenburg et al. 2002). The potential microhabitat difference inferred from the diets is further supported from field observations of pitfall traps that are typically used to sample small terrestrial vertebrate diversity. While both *H. squalostoma* and *G. seraphini* co-occur at sites in Gabon, pitfall traps collected only *G. seraphini* suggesting that it is more active near the surface than *H. squalostoma* (Wollenberg and Measey 2009). Taken together with the results of Jones et al. (2006), our findings suggest that differences in microhabitat use may at least partly drive differences in the prey type consumed by co-occuring caecilian species.

The diets of adult *G. seraphini* and *H. squalostoma* also differ in the number of prey items consumed. Earthworms dominate the diet of adult *G. seraphini*, but *H. squalostoma* consumed more of both earthworms and ants. Adult *G. seraphini* also generally consumed far fewer prey items than *H. squalostoma*. Similar to the differences in prey type discussed above, these differences in prey number may also be consistent with differences in microhabitat. Jones et al. (2006) found that the species (*S. vittatus*) consuming epigeic earthworms ate fewer (and larger) prey items than the sympatric species (*B. boulengeri*) that consumed endogeic earthworms. These observed differences might reflect differences in prey availability at the surface and below, or—and we think this more likely—differences in abundance and variety of soft- vs. hard-bodied prey.

**Fig. 7:**
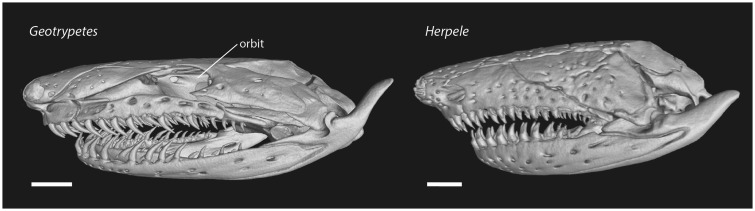
X-ray computed microtomographic (microCT) scans of the adult skull of *Geotrypetes seraphini* (CAS: herp: 259097) and *Herpele squalostoma* (CAS: herp: 258686). Note the orbit and the gracile teeth of *G. seraphini* and the absence of the orbit and the robust teeth of *H. squalostoma.* Scale bar equals 1 mm.

Differences in cranial anatomy between *Herpele* and *Geotrypetes* likely relate directly to the differences observed in the diets. While both species may be opportunistic feeders, the consistent differences in adult diet across our three study sites suggest differences in preferences for soft- vs. hard-bodied prey types. These two species differ in the degree of skull fenestration, reduction, and/or covering of the eye, and the position of the mouth (Sherratt et al. 2014). While variation among species in skull fenestration may not reflect performance differences in burrowing (Kleintech et al. 2012), it might relate to differences in feeding biomechanics such as crushing hard-bodied prey. The skull of *G. seraphini* exhibits temporal fenestration between the squamosal and the parietal (zygokrotaphy; Fig. 7) and orbits that accommodate reduced but externally visible eyes. In contrast, the skull of *H. squalostoma* lacks both fenestration (stegokrotaphy) and orbits (i.e., the eye is completely enclosed within the bony skull, due to expansion of the squamosal and maxilla), and the lower jaw is more distant from the rostrum than in *G. seraphini*. The differences in tooth morphology between *G. seraphini* and *H. squalostoma* also likely relate to the different dominant prey types of each species. In adult *G. seraphini*, the teeth on both the upper and lower jaws are long, thin, and recurved, whereas the teeth of *H. squalostoma* are stout and conical (Fig. 7). In addition, for the specimens examined, *G. seraphini* (mandible, 16 labial, 11 lingual; maxilla, 10 labial, 10 lingual) has more teeth in the lower jaw than *H. squalostoma* (mandible, 11 labial, 2 lingual; upper jaw, 9 lingual, 11 labial). We interpret the gracile tooth morphology of *G. seraphini* as related to piercing and tearing soft-bodied prey such as earthworms and the more robust teeth of *H. squalostoma* as needed for capturing and crushing hard-bodied prey such as mole crickets, beetles, and crabs.

### Diet of juvenile caecilians

The diets of juvenile caecilians are even less well studied than those of adults. In a number of oviparous and at least one viviparous species (*G.seraphini*), young feed on their attending mother’s skin (dermatophagy; Kupfer et al. 2006, 2016; Wilkinson et al. 2008, 2013). Viviparity and uterotrophy, in which offspring feed on intraoviductal secretions (Parker 1956; Exbrayat and Hraoui-Bloquet 1992; Wake and Dickie 1998), are hypothesized to have evolved from skin-feeding (Kupfer et al. 2006). Yet the diversity of prey consumed by juveniles, as well as possible ontogenetic shifts in prey diversity, have not been investigated in depth across caecilian species. We did not detect significant ontogenetic diet shifts for either *G. seraphini* and *H. squalostoma*. This is unsurprising for *G. seraphini* given that both juveniles and adults mostly consume earthworms. We suggest caution, however, in interpreting the non-significance of ontogenetic diets shifts in *H. squalostoma* as this may be due to our small sample sizes*.* Many adults of this species consumed mole crickets which were completely absent in the diets of juveniles of this species. For terrestrial caecilian species, there appears to be a general trend of increased dietary breadth going from juvenile to subadult and adult. In *G.ramaswamii* (family Indotyphlidae), Measey et al. (2004) found that juveniles (*n *=* *5) consumed only termites and earthworms in comparison to subadults and adults (*n *=* *62) that consumed termites, ants, earthworms, beetles, and other arthropods. Similarly, Gaborieau and Measey (2004) report that juvenile *B. taitanus* consumed only earthworms whereas adults eat a variety of prey types including earthworms, termites, tipulid fly larvae, and centipedes. In contrast, the diet of aquatic larvae of *Ichthyophis* cf. *kohtaoensis* (family Ichthyophiidae) contains a broad prey diversity dominated by benthic aquatic arthropods (Kupfer et al. 2005). While the diversity prey types shift ontogenetically as juveniles metamorphose and become terrestrial adults, the diversity of prey types remains broad suggesting that *I.* cf. *kohtaoensis* is a generalist predator as both larvae and adults. Similarly, another study of aquatic larvae in *Typhlonectes compressicauda* (family Typhlonectidae) found a broad range of prey taxa that includes flies, beetles, hemipterans, and both frog eggs and tadpoles, aquatic earthworms, and insects dominated the diet of juveniles (Verdade et al. 2000). Based on the few studies with data for diets of juvenile caecilians, there appears to be a pattern suggesting that terrestrial juveniles have a more limited dietary breadth than aquatic larval caecilians.

*Herpele squalostoma* is an oviparous species in which females are known to attend eggs and provision young through skin feeding (maternal dermatophagy; Kouete et al. 2012, 2013). This form of parental care is best documented in another oviparous herpelid caecilian, *B. taitana* from East Africa. In this species, the attending female loses weight (Kupfer et al. 2008), probably because of provisioning its skin (and associated lipids) to its young and possibly because egg attendance may reduce feeding opportunities for the attending female. In both species, females attend either newly born (*G. seraphini*) or recent hatchlings (*H. squalostoma*) and juveniles as well as provide further maternal investment by offspring engaging in skin-feeding (O’Reilly et al. 1998; Kouete et al. 2012, 2013). Because we observed skin fragments only in juvenile *H. squalostoma* from our samples collected during mid-June to mid-August, the absence of skin fragments in our sample of juvenile *G. seraphini* suggests that these two species may attend and provision young in different seasons. However, we found the diets of juvenile *H. squalostoma* and *G. seraphini* to be largely similar (high overlap, PSI = 0.8). We did not find any juvenile *G. seraphini* attended by another larger individual and all of our samples were fully pigmented. The smallest individual (113 mm in total length) in our sample is much larger than the size reported by Parker and Dunn (1964) for newborn *G. seraphini* (from Sierra Leone), which ranged from 73 to 77 mm total length. Our sample indicates that skin-feeding ceases in *G. seraphini* by ∼110 mm in total length and that egg attendance and maternal provisioning occur at a different time of year than in *H. squalostoma*. In contrast, we sampled one subadult *H. squalostoma* measuring 180 mm total length that contained skin fragments. This suggests that *H. squalostoma* offspring may have a more prolonged period of maternal provisioning than *G. seraphini*, though our results also indicate that juvenile *H. squalostoma* do not only feed on skin while they are attended by the mother. Juvenile *G. seraphini* are generally longer and heavier than juvenile *H. squalostoma*. Whereas the diets of juvenile and adult *G. seraphini* are similar, the diet of *H. squalostoma* becomes more diverse and dominated by a new prey item (mole crickets) as individuals transition from juvenile to adulthood.

### Summary

Whereas previous work by Jones et al. (2006) suggests dietary differences between sympatric caecilian species is driven by microhabitat, our study suggests differences based on specialization for soft- or hard-bodied prey type. To further test potential preferences in prey type, caecilian dietary studies need to incorporate sampling of prey abundance and variation across sites. While ours is only the second study of diets of sympatric caecilian species, we suspect that future work may find similar differences in diet driven by a combination of microhabitat and specialization on soft- or hard-bodied prey.

## Supplementary Material

obz035_Supplementary_Data
