# Supplementary material for: Dietary Partitioning in Two Co-occurring Caecilian Species (Geotrypetes seraphini and Herpele squalostoma) in Central Africa
Source: Integr Org Biol. 2019 Dec 31;2(1):obz035. doi: 10.1093/iob/obz035 (PMC7671121; doi:10.1093/iob/obz035)
Supplement: obz035_Supplementary_Data [file obz035_supplementary_data.zip › MS_revision_IOB_anon_notables_Marcel27Nov19_DCB.docx]

**Abstract.—**Trophic interactions among fossorial vertebrates remain poorly explored in tropical ecosystems. While caecilian species can co-occur, whether and how sympatric species partition dietary or other resources are largely unknown. Based on specimens collected during field surveys in southern Cameroon, we conducted a dietary analysis of two co-occurring caecilian species, *Geotrypetes seraphini* and *Herpele squalostoma*. We find a negligible overlap in the adult diets of these two species. Earthworms dominated the diet of adult *G. seraphini*, whereas we found that mole crickets were the most frequent prey items in adult *H. squalostoma*. The dietary breadth of adult *G. seraphini* is smaller than that of *H. squalostoma*, which consumes a variety of hard-bodied prey including mole crickets, cockroaches, beetles, and crabs. Juvenile diets were similar between these species and mostly contained earthworms and ants. We did not detect significant ontogenetic dietary shifts in either species, though adults generally consumed a broader diversity of prey. As adults, *G. seraphini* and *H. squalostoma* may partition prey categories by consuming soft-bodied and hard-bodied prey, respectively. Because most caecilians are likely opportunistic predators, we expect that sympatric species partition dietary resources either by preference for different soil layers or ability to consume different prey categories.

*Key Words*. Cameroon; Dermophiidae; fossoriality; Gryllotalpidae; Gymnophiona; Herpelidae; Oligochaeta; ontogeny.

Among burrowing vertebrates, the ecology of caecilians—limbless and tropical amphibians—remains poorly studied (Wells, 2007). Adults of most of the 213 species of caecilians are terrestrial, occupying leaf litter and soils in the tropics of Africa, Central and South America, and southern Asia, and even oceanic islands such as São Tomé, the Seychelles, and the Philippines (Gower and Wilkinson, 2008; AmphibiaWeb, 2019). In addition, the free-living and feeding larvae of many caecilians are aquatic as are both larvae and adults of the South American Typhlonectidae. Many caecilians exhibit derived life histories and reproductive modes, including viviparity, direct development, extended parental care, and, perhaps most famously, dermatophagy, in which neonates are attended by the mother and feed on her skin (Wake and Dickie, 1995; Wells, 2007; Kupfer et al., 2008). Relative to salamanders and frogs, little is known of the diets of juvenile and adult caecilians. Most published accounts have limited sampling of intraspecific variation, including studies with just one or several individuals with no information on variation among individuals, sexes, or ontogenetic stages (e.g., Barbour and Loveridge, 1928; Moll and Smith, 1967; Presswell et al., 2002). Studies of caecilian diets that incorporate intraspecific variation are relatively recent, beginning largely with a series of quantitative studies on *Boulengerula taitana* (Gaborieau and Measey, 2004), *Gegeneophis ramaswamii* (Measey et al., 2004) and *Schistometopum thomense* (Delêtre and Measey, 2004). These works provide insights into the trophic niche breadth in caecilians and also demonstrate that these poorly known vertebrates are generalist predators of soil and terrestrial invertebrates (Gaborieau and Measey, 2004).

While there are fewer than a dozen caecilian species for which intraspecific variation in diet has been studied, the dietary breadths of these species are fairly similar. Most studies reveal that caecilian diets are dominated by earthworms and aquatic or terrestrial soil insects such as termites and ants (e.g., Barbour and Loveridge, 1928; Wake, 1980; Verdade et al., 2000; Measey et al*.*, 2004; Kupfer et al., 2005). Some species are known to consume hard-bodied invertebrate prey, including snails (Ngo et al., 2014) and crabs (Gudynas et al., 1988), and occasionally vertebrates such as scolecophidian snakes (Presswell et al., 2002), lizards (Moll and Smith, 1967), and frogs (Gudynas et al., 1988; Kupfer et al., 2005). Hebrard et al. (1992) suggested that caecilians may be at least partially detritivorous, though later authors have rejected this (Gaborieau and Measey, 2004; Delêtre and Measey, 2004).

Most caecilians are thought to be dietary generalists with life history, ecology (i.e., aquatic vs. terrestrial), and seasonal changes in local prey abundance all driving variation within and among species (Kupfer et al., 2005; Ngo et al., 2014). Some caecilian species may specialize on particular prey types, including *Caecilia gracilis* (Maciel et al., 2012) and *Schistometopum thomense* (Delêtre and Measey, 2004) which are both thought to specialize on earthworms. Because most studies sampled few individuals and neither prey abundance nor variation across sites or seasons is typically investigated, it is difficult to disentangle whether a species is a specialist or is instead an opportunist feeding on locally abundant prey types. In addition, almost nothing is known of the diets of caecilian species in sympatry, including whether co-occurring species might eat different prey. In the only study to address this issue, Jones et al. (2006) found differences in the diets of sympatric *Scolecomorphus vittatus* and *Boulengerula boulengeri* in Tanzania. While these species are found at the same sites, this study suggested that the two species feed on different types of earthworms related to foraging in different soil layers.

We extend dietary knowledge of caecilians by providing the second dietary study of co-occurring caecilians and the first studies of intraspecific variation between sexes and ontogenetic stages for two Cameroonian caecilian species, *Geotrypetes seraphini* (family Dermophiidae) and *Herpele squalostoma* (family Herpelidae). The caecilian fauna of Cameroon is the most phylogenetically diverse in Africa, comprising seven species in four families, including five endemic species (Wilkinson et al., 2011). In general, the diets of African caecilian species appear similar to that of species from other regions of the world, largely feeding on soil invertebrates (Table 1), but the diets of the diverse Cameroonian fauna have not yet been investigated. We focused our study on the two most common species, *G. seraphini* and *H. squalostoma*, in Cameroon. These species are widely distributed across West and Central Africa and can be especially abundant in cultivated areas such as gardens and plantations. In addition, these species are known to be sympatric in at least some parts of their respective ranges, including in Cameroon (Gower et al., 2014). While sometimes sympatric, the biology of each species is distinct, including in reproductive mode. *Herpele squalostoma* is oviparous with females attending eggs and juveniles, including provisioning young via skin feeding (Kouete et al., 2012, 2013). In contrast, *G. seraphini* is viviparous but also with altricial young provisioned by the attending mother by skin feeding (Parker, 1956; O’Reilly et al., 1998). We conducted field surveys at three sites in Cameroon and examined gut contents of juveniles and adults of both species to (1) characterize the diversity of prey consumed (Delêtre and Measey, 2004; Gaborieau and Measey, 2004; Measey et al., 2004), (2) analyse patterns of intraspecific variation, both between sexes and ontogenetic stages (Kupfer et al., 2005; Jones et al., 2006; Kouete et al., 2012), (3) test whether the dietary breadth and diversity of prey categories differ for these.

Materials and Methods

*Field Surveys.—*We conducted our study at three field sites (Fig. 1) in southern Republic of Cameroon (datum WGS84): Etam (04° 42’ 80.3”N, 09° 32’ 52.7”E), Ndikinimeki (04° 45’ 77.2”N, 10° 48’ 25.9”E), and in Mundame near Meta quarter (04° 33’ 08.5”N, 09° 31’ 35.2”E). The habitat at these sites is modified by agriculture including both cash crops (cacao, coffee, rubber) and food crops (cassava, cocoyam, and other vegetables). We focused our searches near small streams less than two meters wide as well as water seepages that, according to locals, flow only during the rainy season. We conducted our surveys in 2014 from mid-June to mid-August when the weather in Cameroon transitions from the minor rainy season into the minor dry season.

We collected caecilians by digging in soil with hoes and shovels (following Gower et al., 2014). Because stomach flushing is not known to be an effective tool for studying caecilian diets, we collected diet contents after preserving voucher specimens. We euthanized specimens within 24 hours of their capture in an aqueous solution of MS-222 and then weighed (to the nearest g) and measured (total length, to nearest mm) each specimen. A small sample of liver tissue was preserved in RNALater for future genetic studies. We then fixed all specimens in a solution of 10% neutral buffered formalin for ~48 hr, rinsed in water, and transferred them to 70% ethanol for storage. Once preserved, we took additional measurements for each specimen, including head length, head width (taken at the corner of the mouth), lower jaw length, and both width and circumference at mid-body. We used digital callipers to record all body measurements, except for the mid-body circumference which we measured using thread and a ruler (following Wilkinson et al., 2013). Following previous work by Malonza and Measey (2005) on another herpelid species (*Boulengerula taitanus*), we categorized specimens of both *G. seraphini* and *H. squalostoma* into three life stages based on total length: juveniles (< 140 mm; containing neonates and larger individuals), subadults (< 240 mm), and adults (> 240 mm). We determined sex by direct examination of gonads via dissection; in some cases, gonads were not clearly discernable and thus we refrained from categorizing those specimens as male or female. All specimens are catalogued at the California Academy of Sciences (San Francisco, California, USA).

The occurrence and abundance of *G. seraphini* and *H. squalostoma* varied across our three field sites (Etam, Mundame and Ndikinimeki). Two localities (Etam and Ndikinimeki) yielded sympatric populations of *G. seraphini* and *H. squalostoma.* During field sampling, individuals of both species co-occurred at three digging events including one in Etam and two in Ndikinimeki. At Ndikinimeni, we observed individuals as close as 10 cm suggesting that these two species can be closely associated. A total of 67 specimens of both species (including 4 *G. seraphini* and 63 *H. squalostoma*) were collected at Etam*,* whereas 24 others (comprising 21 *G*. *seraphini* and 3 *H*. *squalostoma*) were recorded at Ndikinimeki. Overall, we sampled 107 specimens of *H. squalostoma* (45 females, 38 males, 19 juveniles and 5 indeterminate) and 24 specimens of *G. seraphini* (11 females, 7 males, 5 juveniles and 1 indeterminate). The sex and/or ontogenetic stage could not be determined for several samples that are larger than juveniles (>140 mm) but that could not be identified definitively as either males or females. Statistics for length and mass of individuals of both species are summarized in Table 2 and 3.

*Categorization of Diet.—*We incised preserved specimens ventrally from below the heart to just anterior to the cloaca. We removed and weighed on a Pesola scale the alimentary canal (i.e., gut) and weighed its contents separately (to the nearest 0.0001 g). We sorted gut contents using a dissecting microscope, and then counted and identified individual prey items (generally to the order or family, but when possible to the genus level). When counting, we attempted to avoid overestimating the number of prey that might be represented by multiple fragments, generated in part during feeding when caecilians bite and then spin to tear prey items (Measey and Herrel, 2006). Especially for earthworms, we searched among fragments in the contents of an individual to attempt matches and then treated these co-occurring pieces as a single item. We were unable to identify taxa of earthworms more specifically due to the fragmentary nature of these prey items. Subsequent data, including length and width for whole food items, were recorded using an ocular micrometer with a Leica S6D microscope (10X magnification), aided by a handheld ruler for large prey; we did not record these data for partial or dissolved food items. In cases where partially digested food items were recognizable (i.e., head capsules of ants and orthopterans), we estimated dimensions following Hirai and Matsui (2001). Both direct and estimated measurements of length and width were used to evaluate the volume of food items by applying the equation of ellipsoid bodies (Colli and Zamboni, 1999), $V=\frac{4}{3}\pi\times(\frac{1}{2}\times length)\times{(\frac{1}{2}\times width)}^{2}$. When searching through gut contents of juveniles of both *G. seraphini* and *H. squalostoma*, we carefully inspected various food items to determine whether skin fragments ingested during skin-feeding might be attached or embedded. We unambiguously identified skin fragments by staining these with iodine. The lipids in the skin fragments become a vivid yellow color when exposed to iodine (Wilkinson et al., 2008). Because of the irregularity of their size and shape, we only counted the frequency of skin fragments among specimens rather than counting and measuring individual pieces within an individual. We grouped other measurable items (with values for length and width) into food categories and used these to quantify the diet of both *G. seraphini and H. squalostoma*.

*Statistical Analyses*.—We evaluated both the frequency and abundance (taking into account the number and the volume) of food found in the gut of specimens of each species and at different life stages. To determine the relative importance of each food category, we calculated the index of relative importance $(IRI)$ following Pinkas et al. (1971) for juveniles and both males and females (for subadults and adults combined) of each species. When calculating *IRI*, we considered only individuals in which the gut contained at least one prey item; individuals with empty guts were excluded from analyses. For any food category $i$, we calculated $IRI$ as follow:

${IRI}_{i}=({NP}_{i}+{VP}_{i})\times{FP}_{i}$, where ${FP}_{i}$ is the percentage of occurrence of food items category $i$ (100 $\times$number of individuals containing food items category $i$/total number of individuals), ${NP}_{i}$ is the percentage abundance (100 $\times$ total number of food items $i$ contained in guts of all individuals / total number of items for all food categories contained in all individuals), and ${VP}_{i}$ is the volumetric percentage 100$\times$total volume of food items category $i$ in guts of all individuals /total volume of all food categories in all individuals).

To make comparisons among species, sex, and ontogenetic stages, we calculated dietary breadth ($B$) following Levins (1968): $=\frac{1}{\sum_{N} P_{j}^{2}}$ , where $P_{j}$ is the numerical proportion of prey category $j$ in the diet and $N$ is the total number of prey categories. Values of $B$ range from 1 to $N$ depending on whether only one prey category or all food categories occurred in a group of individuals.

We also evaluated the extent of overlap in diet among species, sexes, and ontogenetic stages. We calculated an index of overlap, $PS$, proposed by Schoener (1968). For specimens belonging to group $i$, $PS$ was determined as: ${PS}_{i}=1-0.5\sum_{j} |P_{ij}-q_{j}|$,

where $P_{ij}$ is the numerical proportion of food category $j$ in group $i'$s diet, and $q_{j}$ is the proportion of diet category $j$ recorded in all animal groups considered. Values of $PS$ range from 0 (no overlap) to 1 (complete overlap).

Because variation within a species can complicate comparisons between species (Sevenster and Buton, 1998), we took several approaches to investigate variation in diet among individuals. First, we used Mantel tests to examine the extent of diet both between the two species and between subadults and adults (considered together) and juveniles within each species. To compute the Mantel test (a non-parametric test that evaluates correlation among two matrices), we followed Luo and Fox (1996) because their method addresses several challenges inherent to analysis of dietary data, such as unequal sampling sizes within and among species as well as abundances and frequencies that are often aggregated (Anderson, 2001; see Guillot and Rousset, 2013 for possible biases of simple and partial Mantel tests). This method requires construction of a distance matrix that represents the overlap of diet between two groups and a second matrix that represents the null hypothesis of perfect segregation of diet between the two groups. We calculated the distance matrix from the proportion of food items in the gut of each individual in each group compared. For this, we averaged food categories across all individuals by dividing the volumetric proportions by the total volume of each prey category (Bolnick et al., 2002). We used these proportions to calculate the Manhattan distance, an index relative to the proportional similarity measure (see Luo and Fox, 1996). We slightly modified this matrix of similarities to correct for unequal sample size that provides more power to our test (following Luo and Fox, 1996). All Mantel tests were computed using the *ape* package (Paradis et al., 2004) in R version 3.3.2 (R Core Team, 2016) with each analysis set to run for 1000 permutations. To investigate differences in diet that may be due to differences in habitat type (coffee vs. food-crop farms), we used the Fisher exact test for chi-square. Last, we used ANCOVA to investigate differences of gut content mass (dependent variable) that may be due to sex (interactions males/females x gut content mass). Gut content mass for each specimen was calculated as a sum of the masses of individual prey items consumed. We used the cor.test function to explore relationships between the number of prey or their size (length and width) and body attributes in adult *G. seraphini* and *H. squalostoma*.

To perform ANCOVAs, we determined the best predictor for gut content mass from a subset of the body attributes recorded for each species (total length, head length, head width, lower jaw length, mid-body width, mid-body circumference). We used this approach so as to analytically choose the independent variable for ANCOVA rather than choosing one based on other assumptions (e.g., Jones et al., 2006; Measey et al., 2004). Using the set of predictor variables, we constructed a generalized linear model and performed a multi-model selection using the “dredge” function in R’s MuMIn package (Bartoń, 2012). This function constructs and fits all possible candidate sub-models nested within the global model (comprising all recorded predictors), and then ranks them according to either model averaging or any other specified information criteria.). We used the Bayesian Information Criterion (BIC) to rank our candidate models (Johnson and Omland, 2004). We considered models less than 3 BIC units to be similar, and in such cases chose the model with the fewest parameters or the lowest BIC. For cases in which the best models with the dredging approach comprised more than one covariate, we additionally performed ANOVA to pick the ultimate best predictor for gut content mass.

We assessed data normality by applying the Shapiro test. We natural-log transformed gut content mass—the dependent variable in our analysis—because it did not meet the assumption of normality. We further compared the diets of female and male *G. seraphini* and *H. squalostoma* by assessing the relationships of the independent variable for each species as a function of log-transformed values of gut content mass. We used a significance cut-off of 0.05 for statistical tests, all of which we performed in R (R Core Team, 2016).

Results

*Gut Contents*.—Our analysis of gut contents produced ten prey categories (Table 4) that differ in their frequency, abundance, and/or volume across species, life stage, and sex (Table 5). The dominant prey category differs between species for adults and subadults (Table 4), mostly due to the amount of earthworms and crickets consumed by each species. Earthworms constituted the most frequent (54%) and most abundant (54%) prey in the diet of *G. seraphini* whereas mole crickets were the most frequent prey (42%) and ants the most abundant (34%) in the diet of *H. squalostoma*. Most specimens of *H. squalostoma* contained a single cricket. In contrast to *H. squalostoma*, the cricket consumption of *G. seraphini* was low (8%). Instead, the most important prey category for *G. seraphini* was earthworms, representing 80% of all prey volume consumed by this species. Earthworms dominate the diet of adult *G. seraphini*, but *H. squalostoma* consumed significantly more of both earthworms and ants (**𝜒**^2^ = 0.29, *P* < 0.001, df = 756, two-tailed test). Total number of prey categories consumed also differs between the species, with *G. seraphini* consuming fewer prey types than *H. squalostoma*. The more taxonomically diverse diet of *H. squalostoma* leads to a larger dietary breadth (4) in comparison to *G. seraphini* (2.5), as well as low overlap between adults of these two species (*PS =* 0.31 and *PS* = 0.47 using prey volume and prey frequencies, respectively). The Mantel test indicate that there is a significant low overlap (observed = 34; two-tailed *t*-test *P* = 0.003) between adults of *G. seraphini* and *H. squalostoma*.

The dredging approach to select the best predictor of gut mass resulted in different predictors for each species. For *G. seraphini*, the best model was within 3 BIC units of three additional models, but was selected due to containing the fewest covariates (Supplementary Table 1) of which mid-body circumference was selected as the best predictor using ANOVA (Supplementary Table 2). For *H. squalostoma*, the best model was within 3 BIC units of three additional models, but was selected because it contained just one covariate, total length, and had the lowest BIC (Supplementary Table 3).

*Sex-based Dietary Differences*.—In *H. squalostoma*, neither size nor sex influenced prey type. While some prey items occurred in only one sex, there were no significant differences between males and females in overall volumetric proportions of prey items consumed by males and females (Fig. 2) or dietary breadths (females 3.6, and males 3.3; *PS* = 0.7). In general, there was a significant and positive relationship for both sexes between body size and prey mass (Fig. 3, Table 6), and the interaction of sex*size was not significant (*F*_1, 69_ = 1.95, *P* = 0.17, Table 7). Five (one male and four female) specimens of *H. squalostoma* contained only a small amount, if any, of prey (≤ 0.001 g). These four females were of an adult size (> 240 mm) typically associated with high levels of prey consumption (see above). One was found attending a litter of five young and a second (with 0.051 g of prey items) was found brooding a clutch of eggs.

Male and female *G. seraphini* had similar diets with earthworms dominating the diets of both sexes (Fig. 4). As with *H. squalostoma*, prey consumption was explained by body size. Neither sex nor the interaction of sex*size were significant, although this may change with greater sample sizes (Tables 6, 8; Fig. 5).

*Diets of Juvenile Caecilians*.—Juvenile specimens of *G. seraphini* (*n* = 5) and *H. squalostoma* (*n* = 19) contained only two prey categories: ants and earthworms (Table 9). Ants were encountered more frequently across individuals and were the most abundant prey item, whereas earthworms represented the greatest volume (Table 9). This pattern is consistent for juveniles both within and between these two species. Juveniles of both species exhibited similar dietary breadths (1.5 for *G. seraphini*, 2 for *H. squalostoma*) and their diets largely overlapped (overlap of 0.8).

Juvenile diets of the two species differed primarily in the presence of skin (Fig. 6). Most (53%) juvenile *H. squalostoma* contained skin fragments with some (21%) containing only skin fragments, most likely reflecting dermatophagy while attended by mothers. Specimens of *H. squalostoma* that contained skin fragments varied in size from 103–180 mm in length. The largest specimen (180 mm) that contained skin fragments is larger than the length at which we considered specimens to be adult, and contained only a large mass of skin fragments (mass = 0.018 g) in its gut. The smallest juvenile specimens of *H. squalostoma* (94 and 97 mm in length, respectively) did not contain skin fragments, but contained earthworms or both earthworms and ants. One juvenile *H. squalostoma* contained skin fragments as well as earthworms and ants.

*Ontogenetic Dietary Analysis.—*Earthworms dominated the diets of both juvenile and adult *G. seraphini*. The diet of adults differed from juveniles by adding both crickets and invertebrate larvae to the juvenile diet of ants and earthworms, as well as adding larger preys. The adult diets included considerably larger earthworms; the maximum earthworm size recorded for adults was 35 mm and 3 mm, for length and width respectively, in contrast to 12.2 mm and 2 mm in juveniles. Adult *G. seraphini* consumed fewer and larger prey items than juveniles, with body length being negatively correlated with prey number (Table 10). The maximum number of prey items in a single juvenile *G. seraphini* was five (all ants) but the maximum recorded in an adult was only three (ant, cricket, and invertebrate larva). Mean prey volume increased from juvenile to adult, whereas mean prey number decreased (Tables 4, 5). We did not detect a significant relationship between any body attributes (length, head width, head length, and lower jaw length) and prey size (length and width) (Table 10). A Mantel test revealed no statistically significant ontogenetic diet shift for *G. seraphini* (observed = 12; two-tailed *t*-test *P* = 0.11).

The dietary shift from juvenile to adult *H. squalostoma* is similar to that observed in *G. seraphini*. The number of prey categories increased from two in juveniles to seven in adult *H. squalostoma*, including several hard-bodied prey that dominated the diet of adults such as beetles, cockroaches, crabs, and crickets. The most abundant prey in both females and males were invertebrate eggs and ants (Table 4). For both sexes, mole crickets were both the most frequently encountered in females and males (84.2% and 74.3%, respectively) and comprised the largest prey volume (90.5 and 64.2%; Table 4). Crickets may be among the first prey categories added to the juvenile diet in *H. squalostoma*; the smallest specimen with a cricket was 185 mm long. Mean prey volume increased from juvenile to adult as did, in contrast to *G. seraphini*, mean prey number (Table 5).There is a positive significant correlation between prey size (length and width) and body attributes in juvenile and adult *H. squalostoma* (Table 10). A Mantel test revealed no statistically significant ontogenetic diet shift for *H. squalostoma* (observed = 87.7; two-tailed *t*-test *P* = 0.56).

Discussion

*Diet of Sympatric Caecilians.—*Our work reveals important differences in the diets of two co-occurring Central African caecilians. Adults of both *G. seraphini* and *H. squalostoma* consumed earthworms, ants, crickets, and various invertebrate larvae. However, the relative importance of these prey categories and the dietary breadth differed with the diet of *G. seraphini* being relative narrow (breadth = 1.5) and dominated by earthworms and that of *H. squalostoma* being substantially broader and dominated by mole crickets (breadth = 3.5). The greater dietary breadth of *H. squalostoma* includes six additional prey categories (invertebrate eggs, beetles, cockroaches, crabs, earwigs, and termites) and contributes to the low but still significant overlap in diet (*PSI* = 0.46) between these two species. A previous study by Jones et al. (2006) on two caecilian species that occur in sympatry in East Africa (at Nilo Forest Reserve in Tanzania) found similar patterns related to breadth and relative importance of difference. In this case, *Scolecomophus vittatus* (family Scolecomorphidae) had a relatively narrow diet in comparison to the co-occuring *B. boulengeri* (family Herpelidae). Earthworms dominate the diets of both species, though *S. vittatus* consumed mostly large, pigmented epigeic species and *B. boulengeri* consumed smaller, unpigmented endogeic species. While Jones et al. (2006) did not calculate statistics for dietary breadth and overlap, it is clear that in both our study and theirs that the sympatric caecilian species differ in both breadth and prey categories.

The different diets of adult *G. seraphini* and *H. squalostoma* are suggestive of partitioning dietary resources by microhabitat. First, earthworms consumed by *G. seraphini* are large and pigmented (typical of epigeic taxa) suggesting that this species forages at or near the surface whereas the mole crickets that dominate the diet of *H. squalostoma* suggest this species forages underground. Mole crickets are active burrowers that create tunnels in which they lay and guard eggs (Bennet-Clark, 1987), and often considered agricultural pests (Brandenburg et al., 2002). The potential microhabitat difference inferred from the diets is further supported from field observations of pitfall traps that are typically used to sample small terrestrial vertebrate diversity. While both *H. squalostoma* and *G. seraphini* co-occur at sites in Gabon, pitfall traps collected only *G. seraphini* suggesting that it is more active near the surface than *H. squalostoma* (Wollenberg and Measey, 2009). Taken together with the results of Jones et al. (2006), our findings suggest that differences in microhabitat use may at least partly drive differences in the prey type consumed by co-occuring caecilian species.

The diets of adult *G. seraphini* and *H. squalostoma* also differ in the number of prey items consumed. Earthworms dominate the diet of adult *G. seraphini*, but *H. squalostoma* consumed more of both earthworms and ants. Adult *G. seraphini* also generally consumed far fewer prey items than *H. squalostoma*. Similar to the differences in prey type discussed above, these differences in prey number may also be consistent with differences in microhabitat. Jones et al. (2006) found that the species (*S. vittatus*) consuming epigeic earthworms ate fewer (and larger) prey items than the sympatric species (*B. boulengeri*) that consumed endogeic earthworms. These observed differences might reflect differences in prey availability at the surface and below, or—and we think this more likely—differences in abundance and variety of soft- vs. hard-bodied prey.

Differences in cranial anatomy between *Herpele* and *Geotrypetes* likely relate directly to the differences observed in the diets. While both species may be opportunistic feeders, the consistent differences in adult diet across our three study sites suggest differences in preferences for soft- vs. hard-bodied prey types. These two species differ in the degree of skull fenestration, reduction and/or covering of the eye, and the position of the mouth (Sherratt et al., 2014). While variation among species in skull fenestration may not reflect performance differences in burrowing (Kleintech et al., 2012), it might relate to differences in feeding biomechanics such as crushing hard-bodied prey. The skull of *G. seraphini* exhibits temporal fenestration between the squamosal and the parietal (zygokrotaphy; Fig. 7) and orbits that accommodate reduced but externally visible eyes. In contrast, the skull of *H. squalostoma* lacks both fenestration (stegokrotaphy) and orbits (i.e., the eye is completely enclosed within the bony skull, due to expansion of the squamosal and maxilla), and the lower jaw is more distant from the rostrum than in *G. seraphini*. The differences in tooth morphology between *G. seraphini* and *H. squalostoma* also likely relate to the different dominant prey types of each species. In adult *G. seraphini*, the teeth on both the upper and lower jaws are long, thin, and recurved, whereas the teeth of *H. squalostoma* are stout and conical (Fig. 7). In addition, for the specimens examined, *G. seraphini* (mandible, 16 labial, 11 lingual; maxilla, 10 labial, 10 lingual) has more teeth in the lower jaw than *H. squalostoma* (mandible, 11 labial, 2 lingual; upper jaw, 9 lingual, 11 labial). We interpret the gracile tooth morphology of *G. seraphini* as related to piercing and tearing soft-bodied prey such as earthworms and the more robust teeth of *H. squalostoma* as needed for capturing and crushing hard-bodied prey such as mole crickets, beetles, and crabs.

*Diet of Juvenile Caecilians.—*The diets of juvenile caecilians are even less well studied than those of adults. In a number of oviparous and at least one viviparous species (*Geotrypetes seraphini*), young feed on their attending mother’s skin (dermatophagy; Kupfer et al., 2006, 2016; Wilkinson et al., 2008, 2013). Viviparity and uterotrophy, in which offspring feed on intraoviductal secretions (Parker, 1956; Exbrayat and Hraoui-Bloquet, 1992; Wake and Dickie, 1998), are hypothesized to have evolved from skin-feeding (Kupfer et al., 2006). Yet the diversity of prey consumed by juveniles, as well as possible ontogenetic shifts in prey diversity, have not been investigated in depth across caecilian species. We did not detect significant ontogenetic diet shifts for either *G. seraphini* and *H. squalostoma*. This is unsurprising for *G. seraphini* given that both juveniles and adults mostly consume earthworms. We suggest caution, however, in interpreting the non-significance of ontogenetic diets shifts in *H. squalostoma* as this may be due to our small samples sizes*.* Many adults of this species consumed mole crickets which were completely absent in the diets of juveniles of this species. For terrestrial caecilian species, there appears to be a general trend of increased dietary breadth going from juvenile to subadult and adult. In *Gegeneophis ramaswami* (family Indotyphlidae), Measey et al. (2004) found that juveniles (*n* = 5) consumed only termites and earthworms in comparison to subadults and adults (*n* = 62) that consumed termites, ants, earthworms, beetles, and other arthropods. Similarly, Gaborieau and Measey (2004) report that juvenile *B. taitanus* consumed only earthworms whereas adults eat a variety of prey types including earthworms, termites, tipulid fly larvae, and centipedes. In contrast, the diet of aquatic larvae of *Ichthyophis* cf. *kohtaoensis* (family Ichthyophiidae) contains a broad prey diversity dominated by benthic aquatic arthropods (Kupfer et al., 2005). While the diversity prey types shifts ontogenetically as juveniles metamorphose and become terrestrial adults, the diversity of prey types remains broad suggesting that *I.* cf. *kohtaoensis* is a generalist predator as both larvae and adults. Similarly, another study of aquatic larvae in *Typhlonectes compressicauda* (family Typhlonectidae) found a broad range of prey taxa that includes flies, beetles, hemipterans, and both frog eggs and tapdoles, aquatic earthworms and insects dominated the diet of juveniles (Verdade et al., 2000). Based on the few studies with data for diets of juvenile caecilians, there appears to be a pattern suggesting that terrestrial juveniles have a more limited dietary breadth than aquatic larval caecilians.

*Herpele squalostoma* is an oviparous species in which females are known to attend eggs and provision young through skin feeding (maternal dermatophagy; Kouete et al., 2012, 2013). This form of parental care is best documented in another oviparous herpelid caecilian, *B. taitana* from East Africa. In this species, the attending female loses weight (Kupfer et al., 2008), probably because of provisioning its skin (and associated lipids) to its young and possibly because egg attendance may reduce feeding opportunities for the attending female. In both species, females attend either newly born (*G. seraphini*) or recent hatchlings (*H. squalostoma*) and juveniles as well as provide further maternal investment by offspring engaging in skin-feeding (O’Reilly et al., 1998; Kouete et al., 2012, 2013). Because we observed skin fragments only in juvenile *H. squalostoma* from our samples collected during mid-June to mid-August, the absence of skin fragments in our sample of juvenile *G. seraphini* suggests that these two species may attend and provision young in different seasons. However, we found the diets of juvenile *H. squalostoma* and *G. seraphini* to be largely similar (high overlap, *PSI*= 0.8). We did not find any juvenile *G. seraphini* attended by another larger individual and all of our samples were fully pigmented. The smallest individual (113 mm in total length) in our sample is much larger than the size reported by Parker and Dunn (1964) for newborn *G. seraphini* (from Sierra Leone), which ranged from 73–77 mm total length. Our sample indicates that skin-feeding ceases in *G. seraphini* by ~110 mm in total length and that egg attendance and maternal provisioning occur at a different time of year than in *H. squalostoma*. In contrast, we sampled one subadult *H. squalostoma* measuring 180 mm total length that contained skin fragments. This suggests that *H. squalostoma* offspring may have a more prolonged period of maternal provisioning than *G. seraphini*, though our results also indicate that juvenile *H. squalostoma* do not only feed on skin while they are attended by the mother. Juvenile *G. seraphini* are generally longer and heavier than juvenile *H. squalostoma*. Whereas the diets of juvenile and adult *G. seraphini* are similar, the diet of *H. squalostoma* becomes more diverse and dominated by a new prey item (mole crickets) as individuals transition from juvenile to adulthood.

*Summary.—*Whereas previous work by Jones et al. (2006) suggests dietary differences between sympatric caecilian species is driven by microhabitat, our study suggests differences based on specialization for soft- or hard-bodied prey type. To further test potential preferences in prey type, caecilian dietary studies need to incorporate sampling of prey abundance and variation across sites. While ours is only the second study of diets of sympatric caecilian species, we suspect that future work may find similar differences in diet driven by a combination of microhabitat and specialization on soft- or hard-bodied prey.

*Acknowledgments*.—We thank the Cameroonian Ministry of Forests and Wildlife (MINFOF) and Ministry of Scientific Research and Innovation (MINRESI) for permissions necessary to conduct our research. We also thank the local assistants (E. Pasto, C. Ewane, and J. Bouayehok) who assisted MTK during fieldwork. The Institutional Animal Care and Use Committee at the California Academy of Sciences approved our field survey and collection methods (IACUC #2014-2). MTK was supported by a grant from the Lakeside Foundation via the California Academy of Sciences. Field research was supported by a grant from the US National Science Foundation (DEB-1202609) to DCB. We are especially grateful to M. Wake and V. Vredenburg who both provided encouragement and guidance to MTK during this project. Several reviewers provided constructive comments that improved this manuscript.

Literature Cited

AmphibiaWeb. 2019. AmphibiaWeb: Information on Amphibian Biology and Conservation. Berkeley, USA <http://amphibiaweb.org/>.

Anderson, M. J. 2001. A new method for non-parametric multivariate analysis of variance. Austral Ecology 26:32–46.

Barbour, T., and A. Loveridge. 1928. A comparative study of the herpetological faunae of the Uluguru and Usambara Mountains, Tanganyika Territory, with description of new species. Memoires of the Museum of Comparative Zoology 50:85–265.

Bartoń, K. 2012. Package ‘MuMIn’. Model selection and model averaging base on information criteria. R package version 1.42.1. R Foundation for Statistical Computing, Vienna, Austria.

Bennet-Clark, H. C. 1987. The tuned singing burrow of mole crickets. Journal of Experimental Biology 128:383–409.

Bolnick, D.I., L. H. Yang, J. A. Fordyce, J. M. Davis, and R. Svanbäck. 2002. Measuring individual-level resource specialization. Ecology 83:2936–2941.

Brandenburg, R. L., Y. Xia, and A. S. Schoener. 2002. Tunnel architectures of three species of mole crickets (Orthoptera: Gryllotalpidae). Florida Entomologist 85:383–385.

Colli, G. R., and D. S. Zamboni. 1999. Ecology of the worm-lizard *Amphisbaena alba* in the Cerrado of central Brazil. Copeia 1999:733–742.

Delêtre, M., and G. J. Measey. 2004. Sexual selection vs. ecological causation in a sexually dimorphic caecilian, *Schistometopum thomense* (Amphibia Gymnophiona Caeciliidae). Ethology Ecology & Evolution 16:243–253.

Exbrayat, J.-M., and S. Hraoui-Bloquet. 1992. La nutrition embryonnaire et les relations foeto-maternelles chez *Typhlonectes compressicaudus* amphibien gymnophione vivipare. Bulletin de la Société herpétologique de France 61:53–61.

Gaborieau, O., and G. J. Measey. 2004. Termitivore or detritivore? A quantitative investigation into the diet of the East African caecilian *Boulengerula taitanus* (Amphibia: Gymnophiona: Caeciliidae). Animal Biology 54:45–56.

Gower, D. J., M. T. Kouete, T.-M. Doherty-Bone, E. S. Ndeme, and M. Wilkinson. 2014. Rediscovery, natural history, and conservation status of *Idiocranium russeli* Parker, 1936 (Amphibia: Gymnophiona: Indotyphlidae). Journal of Natural History 49:233–253.

Gower, D. J., and M. Wilkinson. 2008. Caecilians (Gymnophiona). Pp. 19–20 in Stuart, S.N., Hoffmann, M., Chanson, J.S., Cox, N.A., Berridge, R., Ramani, P., Young, B.E. (Eds.), Threatened Amphibians of the World. Lynx Ediciones, with IUCN – The World Conservation Union, Conservation International, and NatureServe, Barcelona.

Gudynas, E., J. D. Williams, and M. Azpelicueta. 1988. Morphology, ecology and biogeography of the South American caecilian *Chthonerpeton indistinctum* (Amphibia: Gymnophiona: Typhlonectidae). Zoologische Mededelingen 62:5–28.

Guillot, G., and F. Rousset. 2013. Dismantling the Mantel tests. Methods in Ecology and Evolution 4:336–344.

Hebrard, J. J., G. M. O. Maloiy, and D. M. I. Alliangana. 1992. Notes on the habitat and diet of *Afrocaecilia taitana* (Amphibia: Gymnophiona). Journal of Herpetology 26:513–515.

Hirai, T., and M. Matsui. 2001. Diet composition of the Indian rice frog, *Rana limnocharis*, in rice fields of central Japan. Current Herpetology 20:97–103.

Jones, D. T., S. P. Loader, and D. J. Gower. 2006. Trophic ecology of East African caecilians (Amphibia: Gymnophiona), and their impact on forest soil invertebrates. Journal of Zoology (London) 269:117–126.

Johnson, J. B., and K. S. Omland. 2004. Model selection in ecology and evolution. Trends in Ecology and Evolution 19:101– 108.

Kleintech, T., H. M. Maddin, J. Herzen, F. Beckmann, and A. P. Summers. 2012. Is solid always best? Cranial performance in solid and fenestrated caecilian skulls. Journal of Experimental Biology 215:833–844.

Kouete, M., M. Wilkinson, and D. J. Gower. 2012. First reproductive observations for *Herpele* Peters, 1880 (Amphibia: Gymnophiona: Herpelidae): evidence of extended parental care and maternal dermatophagy in *H. squalostoma* (Stutchbury, 1836). ISRN Zoology 2012: doi:10.5402/2012/269690.

Kouete, M. T., E. S. Ndeme, and D. J. Gower. 2013. Further observations of reproduction and confirmation of oviparity in *Herpele squalostoma* (Stutchbury, 1836) (Amphibia: Gymnophiona: Herpelidae. Herpetological Notes 6:583–586.

Kupfer, A., J. Nabhitabhata, and W. Himstedt. 2005. From water into soil: trophic ecology of a caecilian amphibian (Genus *Ichthyophis*). Acta Oecologica 28:95–105.

Kupfer, A., H. Müller, M. M. Antoniazzi, C. Jared, H. Greven, R. A. Nussbaum, and M. Wilkinson. 2006. Parental investment by skin feeding in a caecilian amphibian. Nature 440:926–929.

Kupfer, A., M. Wilkinson, D. J. Gower, H. Müller, and R. Jehle. 2008. Care and parentage in a skin-feeding caecilian amphibian. Journal of Experimental Zoology 309A:460–467.

Kupfer, A., E. Maxwell, S. Reinhard, and S. Kuehnel. 2016. The evolution of parental investment in caecilian amphibians: a comparative approach. Biological Journal of the Linnean Society 119:4–14.

Largen, M. J., P. A. Morris, and D. W. Yalden 1972. Observations on the caecilian *Geotrypetes grandisonae* Taylor (Amphibia Gymnophiona) from Ethiopia. Monitore Zoologico Italiano, Supplemento 4:185–205.

Levins, R. 1968. Evolution in Changing Environments: Some Theoretical Explorations. Princeton University Press, Princeton, N.J.

Luo, J., and B. Fox. 1996. A review of the mantel test in the dietary studies: effect of sample size and inequality of sample sizes. Wildlife Research 23:267–288.

Maciel, A. O., J. O. Gomes, J. C. L. Costa, and G. V. Andrade. 2012. Diet, microhabitat use, and an analysis of sexual dimorphism in Caecilia gracilis (Amphibia: Gymnophiona: Caeciliidae) from a riparian forest in the Brazilian Cerrado. Journal of Herpetology 46:47–50.

Malonza, P. K., and G. J. Measey. 2005. Life history of an African caecilian: *Boulengerula taitanus* Loveridge 1935 (Amphibia Gymnophiona Caeciilidae). Tropical Zoology 18:49–66.

Measey, G. J., D. J. Gower, O. V. Oomen, and M. Wilkinson. 2004. A subterranean generalist predator: diet of the soil-dwelling caecilian *Gegeneophis ramaswamii* (Amphibia; Gymnophiona; Caeciliidae) in southern India. Comptes Rendus Biologies 327:65–76.

Measey, G. J. and A. Herrel 2006. Rotational feeding in caecilians: putting a spin on the evolution of cranial design. Biology Letters 2:485–487.

Moll, E. O., and H. M. Smith. 1967. Lizards in the diet of an American caecilian. Natural History Miscellany 187:1–2.

Natusch, D. J. D., and J. A. Lyons. 2012. Relationships between ontogenetic changes in prey selection, head shape, sexual maturity, and colour in an Australian python (*Morelia viridis*). Biological Journal Linnaean Society 107:269–276.

Ngo, B. V., N. T. Hoang, and C. D. Ngo. 2014. Diet of the Bannan caecilian *Ichthyophis bannanicus* (Amphibia: Gymnophiona: Ichthyophiidae) in the Mekong Delta, Vietnam. Journal of Herpetology 48:506–513.

O’Reilly, J. C., D. Fenolio, L. C. Rania, and M. Wilkinson. 1998. Altriciality and extended parental care in the West African caecilian *Geotryptes seraphini* (Gymnophiona: Caeciliidae). American Zoologist 38: 187A.

Paradis, E., J. Claude, and K. Strimmer. 2004. APE: analyses of phylogenetics and evolution in R language. Bioinformatics 20:289–29.

Parker, H. W. 1956. Viviparous caecilians and amphibian phylogeny. Nature 178:250–252.

Parker, H. W., and E. R. Dunn. 1964. Dentitional metamorphosis in the Amphibia. Copeia 1964: 75­–86.

Pinkas, L., M. S. Oliphant, and I. L. K. Iverson. 1971. Food habits of albacore, bluefin tuna, and bonito in California waters. California Department of Fish and Game Bulletin: Fish Bulletin 152:1–105.

Presswell, B., D. J. Gower, O. V. Oomen, G. J. Measey, and M. Wilkinson. 2002. Scolecophidian snakes in the diets of south Asian caecilian amphibians. Herpetological Journal 12:123–126.

R Core Team. 2016. R: a language and environment for statistical computing. R Foundation for Statistical Computing, Vienna, Austria. ISBN 3-900051-07-0, URL http://www.R-project.org/.

Schoener, T. W. 1968. The *Anolis* lizards of Bimini: resource partitioning in a complex fauna. Ecology 49:704–726.

Sevenster, J. G., and N. Bouton. 1998. The statistical significance of diets and other resource utilization patterns. Netherlands Journal of Zoology 48:267–272.

Sherratt, E., D. J. Gower, C. P. Klingenber, and M. Wilkinson. 2014. Evolution of cranial shape in caecilians (Amphibia: Gymnophiona). Evolutionary Biology 41:528–545.

Verdade, V. K., L. C. Schiesari, and J. A. Bertoluci. 2000. Diet of juvenile aquatic caecilians, *Typhlonectes compressicauda*. Journal of Herpetology 34:291–293.

Wake, M. H. 1980. Reproduction, growth, and population structure of the Central American caecilian *Dermophis mexicanus*. Herpetologica 36:244–256

Wake, M. H., and R. Dickie. 1998. Oviduct structure and function and reproductive modes in amphibians. Journal of Experimental Zoology 282:477–506.

Wells, K. D. 2007. The Ecology and Behavior of Amphibians. The University of Chicago Press, Chicago.

Wilkinson, M., A. Kupfer, R. Marques-Porto, H. Jeffkins, M. M. Antoniazzi, and C. Jared. 2008. One hundred million years of skin feeding? Extended parental care in a Neotropical caecilian (Amphibia: Gymnophiona). Biology Letters 4:358–361.

Wilkinson, M., D. San Mauro, E. Sherratt, and D. J. Gower. 2011. A nine-family classification of caecilians (Amphibia: Gymnophiona). Zootaxa 2874:41–64.

Wilkinson, M., E. Sherratt, F. Starace, and D. J. Gower. 2013. A new species of skin-feeding caecilian and the first report of reproductive mode in *Microcaecilia* (Amphibia: Gymonphiona: Siphonopidae). PLoS One 8:e57756.

Wollenberg, K. C., and J. G. Measey. 2009. Why colour in subterranean vertebrates? Exploring the evolution of colour patterns in caecilian amphibians. Journal of Evolutionary Biology 22:1046–1056.

Fig. 1. Distribution of the localities (Etam, Mundame and Ndikinimeki) in Cameroon where caecilian specimens were sampled for this study.

Fig. 2. Prey categories consumed by female and male *Herpele squalostoma*.

Fig. 3. Regression of mass of gut content (in g) as a function of total length (TL) (in mm) between female and male *Herpele squalostoma*. Mass of gut content was natural log-transformed to meet the assumption of normality.

Fig. 4. Prey categories consumed by adult *G. seraphini* and *H. squalostoma*.

Fig. 5. Regression of mass of gut content (in g) as a function of MBC (in mm) between female and male *Geotrypetes seraphini*. Mass of gut content was natural log-transformed to meet the assumption of normality.

Fig. 6. Food categories consumed by juveniles of *G. seraphini* and *H. squalostoma*, represented as numerical proportion of total food categories. Note the inclusion of skin fragments in the diet of juveniles of *H. squalostoma* alters the percentages of food items reported in Table 9, which includes only prey items.

Fig. 7. X-ray computed microtomographic (microCT) scans of the adult skull of *Geotrypetes seraphini* (CAS:herp:259097) and *Herpele squalostoma* (CAS:herp:258686). Note the orbit and the gracile teeth of *G. seraphini* and the absence of the orbit and the robust teeth of *H. squalostoma.* Scale bar equals 1 mm.
