# Supplementary material for: Dietary Partitioning in Two Co-occurring Caecilian Species (Geotrypetes seraphini and Herpele squalostoma) in Central Africa
Source: Integr Org Biol. 2019 Dec 31;2(1):obz035. doi: 10.1093/iob/obz035 (PMC7671121; doi:10.1093/iob/obz035)
Supplement: obz035_Supplementary_Data [file obz035_supplementary_data.zip › Supplementary table 1_v2.docx]

Supplementary Table 1. Summary of global model displaying set of candidate models for *G. seraphini* obtained from the “dredge” function of the MUMIN package in R (Barton, 2012). The “dredge” procedure was conducted using log-transformed values of gut content mass as the dependent variable. The independent variable comprised the following body attributes: mid body circumference (MBC), head length (HL), lower jaw length (LJL) mid body width (MBW), head width (HW), and animal total length (TL). Models were ranked using the Bayesian Information Criterion (BIC). The best model is in bold.

| MBC | HL | LJL | MBW | HW | TL | Intercept | Df | logLik | BIC | ∆BIC | Weight |
| --- | --- | --- | --- | --- | --- | --- | --- | --- | --- | --- | --- |
| **x** |  | **x** |  |  | **x** | **-2.03** | **5** | **-22.07** | **58.31** | **0.00** | **0.268** |
| x | x | x |  |  | x | -2.93 | 6 | -21.27 | 59.53 | 1.22 | 0.146 |
| x |  | x |  | x | x | -3.08 | 6 | -21.28 | 59.56 | 1.25 | 0.144 |
| x |  | x | x |  | x | -2.23 | 6 | -22.02 | 61.05 | 2.73 | 0.068 |
| x | x | x | x |  | x | -3.53 | 7 | -21.00 | 61.83 | 3.51 | 0.046 |
| x | x | x |  | x | x | -3.27 | 7 | -21.05 | 61.92 | 3.61 | 0.044 |
| x |  | x |  |  |  | -2.52 | 4 | -25.31 | 61.96 | 3.65 | 0.043 |
| x |  | x | x | x | x | -3.59 | 7 | -21.08 | 61.99 | 3.67 | 0.043 |
| x |  |  |  |  | x | -5.82 | 4 | -25.74 | 62.82 | 4.51 | 0.028 |
| x |  | x | x |  |  | -3.25 | 5 | -24.52 | 63.21 | 4.90 | 0.023 |
| x | x | x | x | x | x | -3.94 | 8 | -20.73 | 64.12 | 5.81 | 0.015 |
| x |  | x |  | x |  | -2.55 | 5 | -25.31 | 64.79 | 6.48 | 0.010 |
| x | x | x |  |  |  | -2.50 | 5 | -25.31 | 64.79 | 6.48 | 0.010 |
| x | x |  |  |  | x | -4.85 | 5 | -25.56 | 65.29 | 6.98 | 0.008 |
| x |  |  |  |  |  | -6.78 | 3 | -28.45 | 65.40 | 7.08 | 0.008 |
| x |  |  | x |  | x | -6.07 | 5 | -25.68 | 65.53 | 7.22 | 0.007 |
| x |  |  |  | x | x | -6.04 | 5 | -25.73 | 65.63 | 7.32 | 0.007 |
| x | x | x | x |  |  | -3.89 | 6 | -24.37 | 65.75 | 7.43 | 0.007 |
| x |  | x | x | x |  | -3.90 | 6 | -24.39 | 65.78 | 7.46 | 0.006 |
|  | x | x |  |  | x | -6.04 | 5 | -25.86 | 65.89 | 7.58 | 0.006 |
|  | x | x | x |  | x | -3.68 | 6 | -24.67 | 66.35 | 8.03 | 0.005 |
| x | x |  |  |  |  | -4.22 | 4 | -27.52 | 66.38 | 8.07 | 0.005 |
|  |  |  | x |  | x | -4.34 | 4 | -27.58 | 66.49 | 8.18 | 0.004 |
| x |  |  | x |  |  | -7.45 | 4 | -27.71 | 66.74 | 8.43 | 0.004 |
|  |  | x | x |  | x | -1.46 | 5 | -26.43 | 67.04 | 8.72 | 0.003 |
| x | x | x |  | x |  | -2.54 | 6 | -25.31 | 67.62 | 9.31 | 0.003 |
| x | x |  |  | x | x | -5.26 | 6 | -25.33 | 67.65 | 9.34 | 0.003 |
| x |  |  |  | x |  | -5.43 | 4 | -28.16 | 67.66 | 9.34 | 0.003 |
|  |  |  | x |  |  | -5.65 | 3 | -29.70 | 67.90 | 9.59 | 0.002 |
|  |  | x | x | x | x | -3.25 | 6 | -25.49 | 67.99 | 9.67 | 0.002 |
| x | x |  | x |  | x | -5.08 | 6 | -25.54 | 68.08 | 9.77 | 0.002 |
| x |  |  | x | x | x | -6.46 | 6 | -25.66 | 68.32 | 10.00 | 0.002 |
|  | x | x |  |  |  | -5.65 | 4 | -28.52 | 68.37 | 10.06 | 0.002 |
|  | x | x |  | x | x | -6.38 | 6 | -25.74 | 68.47 | 10.16 | 0.002 |
| x | x | x | x | x |  | -4.06 | 7 | -24.34 | 68.52 | 10.21 | 0.002 |
| x | x |  | x |  |  | -5.27 | 5 | -27.21 | 68.58 | 10.27 | 0.002 |
|  | x |  |  |  | x | -8.08 | 4 | -28.74 | 68.80 | 10.49 | 0.001 |
|  |  | x | x |  |  | -2.63 | 4 | -28.75 | 68.83 | 10.52 | 0.001 |
|  | x |  |  |  |  | -7.47 | 3 | -30.17 | 68.85 | 10.54 | 0.001 |
|  |  |  | x | x | x | -5.60 | 5 | -27.39 | 68.95 | 10.63 | 0.001 |
|  | x | x | x | x | x | -4.00 | 7 | -24.57 | 68.97 | 10.66 | 0.001 |
| x | x |  |  | x |  | -4.37 | 5 | -27.50 | 69.16 | 10.84 | 0.001 |
|  |  |  |  | x |  | -7.60 | 3 | -30.33 | 69.16 | 10.85 | 0.001 |
|  | x |  | x |  | x | -5.11 | 5 | -27.52 | 69.21 | 10.90 | 0.001 |
| x |  |  | x | x |  | -6.71 | 5 | -27.64 | 69.44 | 11.13 | 0.001 |
|  |  |  |  | x | x | -8.35 | 4 | -29.13 | 69.60 | 11.28 | 0.001 |
| x | x |  | x | x | x | -5.57 | 7 | -25.29 | 70.41 | 12.10 | 0.001 |
|  | x | x | x |  |  | -4.19 | 5 | -28.21 | 70.58 | 12.26 | 0.001 |
|  | x |  | x |  |  | -5.38 | 4 | -29.70 | 70.73 | 12.41 | 0.001 |
|  |  |  | x | x |  | -5.69 | 4 | -29.70 | 70.74 | 12.42 | 0.001 |
|  |  |  |  |  |  | -2.66 | 2 | -32.56 | 70.79 | 12.47 | 0.001 |
|  |  | x |  | x |  | -6.68 | 4 | -29.81 | 70.96 | 12.65 | 0.000 |
|  | x | x |  | x |  | -5.68 | 5 | -28.52 | 71.21 | 12.89 | 0.000 |
| x | x |  | x | x |  | -5.56 | 6 | -27.14 | 71.28 | 12.97 | 0.000 |
|  | x |  |  | x | x | -8.50 | 5 | -28.57 | 71.31 | 13.00 | 0.000 |
|  |  | x |  | x | x | -7.44 | 5 | -28.57 | 71.31 | 13.00 | 0.000 |
|  |  | x | x | x |  | -3.58 | 5 | -28.58 | 71.33 | 13.01 | 0.000 |
|  | x |  |  | x |  | -7.62 | 4 | -30.15 | 71.64 | 13.33 | 0.000 |
|  | x |  | x | x | x | -5.52 | 6 | -27.39 | 71.78 | 13.46 | 0.000 |
|  |  | x |  |  |  | -5.65 | 3 | -31.90 | 72.30 | 13.98 | 0.000 |
|  |  |  |  |  | x | -3.72 | 3 | -32.32 | 73.15 | 14.83 | 0.000 |
|  | x | x | x | x |  | -4.17 | 6 | -28.21 | 73.41 | 15.10 | 0.000 |
|  | x |  | x | x |  | -5.48 | 5 | -29.69 | 73.55 | 15.24 | 0.000 |
|  |  | x |  |  | x | -5.66 | 4 | -31.90 | 75.13 | 16.81 | 0.000 |
