# Supplementary material for: Dietary Partitioning in Two Co-occurring Caecilian Species (Geotrypetes seraphini and Herpele squalostoma) in Central Africa
Source: Integr Org Biol. 2019 Dec 31;2(1):obz035. doi: 10.1093/iob/obz035 (PMC7671121; doi:10.1093/iob/obz035)
Supplement: obz035_Supplementary_Data [file obz035_supplementary_data.zip › Supplementary table 2_v2.docx]

Supplementary Table 2. ANOVA summary which compared among the variables selected by the dredging approach in the best performing model. For this analysis log transformed values of gut content mass was the dependent variable while mid-body circumference (MBC), lower jaw length (LJL) and total length (TL) were the independent variables This additional steps serves to identify the overall best predictor for *G. seraphini* gut*.* The values for the residual (*e*), collinearity (*R*^2^), and F statistics for the best predictor are in bold.

| Variables | F | *e* | *R^2^* |
| --- | --- | --- | --- |
| MBC | 9.3 | 1.37 | 0.38 |
| LJL | 1.21 | 1.68 | 0.08 |
| TL | 0.42 | 1.73 | 0.03 |
