# Supplementary material for: Dietary Partitioning in Two Co-occurring Caecilian Species (Geotrypetes seraphini and Herpele squalostoma) in Central Africa
Source: Integr Org Biol. 2019 Dec 31;2(1):obz035. doi: 10.1093/iob/obz035 (PMC7671121; doi:10.1093/iob/obz035)
Supplement: obz035_Supplementary_Data [file obz035_supplementary_data.zip › Supplementary Table 4.docx]

Supplementary Table 4. Summary of linear regression for the relationship between gape index as a function of prey width in adults subadults and juveniles of *G. seraphini* and *H. squalostoma*. None of the relationships is significant.

|  |  | Sources | | | |
| --- | --- | --- | --- | --- | --- |
| Species | Life stage/Sex | Df | F | R^2^ | P-value |
| *G. seraphini* | Adults and subadults | 9 | 0.034 | 0.004 | 0.8 |
| *H. squalostoma* | Juveniles | 10 | 2.05 | 0.17 | 0.18 |
|  | Males | 31 | 0.278 | 0.009 | 0.6 |
|  | Females | 35 | 0.16 | 0.0045 | 0.7 |
